# Do Dietary Trajectories between Infancy and Toddlerhood Influence IQ in Childhood and Adolescence? Results from a Prospective Birth Cohort Study

**DOI:** 10.1371/journal.pone.0058904

**Published:** 2013-03-13

**Authors:** Lisa G. Smithers, Rebecca K. Golley, Murthy N. Mittinty, Laima Brazionis, Kate Northstone, Pauline Emmett, John W. Lynch

**Affiliations:** 1 Discipline of Public Health, The University of Adelaide, Adelaide, South Australia, Australia; 2 School of Pharmacy and Medical Sciences, University of South Australia, Adelaide, South Australia, Australia; 3 School of Social and Community Medicine, Bristol University, Bristol, England; Pennington Biomedical Research Center/LSU, United States of America

## Abstract

**Objective:**

We examined whether trajectories of dietary patterns from 6 to 24 months of age are associated with intelligence quotient (IQ) in childhood and adolescence.

**Methods:**

Participants were children enrolled in a prospective UK birth cohort (n = 7652) who had IQ measured at age 8 and/or 15 years. Dietary patterns were previously extracted from questionnaires when children were aged 6, 15 and 24 months using principal component analysis. Dietary trajectories were generated by combining scores on similar dietary patterns across each age, using multilevel mixed models. Associations between dietary trajectories and IQ were examined in generalized linear models with adjustment for potential confounders.

**Results:**

Four dietary pattern trajectories were constructed from 6 to 24 months of age and were named according to foods that made the strongest contribution to trajectory scores; *Healthy* (characterised by breastfeeding at 6 months, raw fruit and vegetables, cheese and herbs at 15 and 24 months); *Discretionary* (biscuits, chocolate, crisps at all ages), *Traditional* (meat, cooked vegetables and puddings at all ages) and, *Ready-to-eat* (use of ready-prepared baby foods at 6 and 15 months, biscuits, bread and breakfast cereals at 24 months). In fully-adjusted models, a 1 SD change in the *Healthy* trajectory was weakly associated with higher IQ at age 8 (1.07 (95%CI 0.17, 1.97)) but not 15 years (0.49 (−0.28, 1.26)). Associations between the *Discretionary* and *Traditional* trajectories with IQ at 8 and 15 years were as follows; *Discretionary*; 8 years −0.35(−1.03, 0.33), 15 years −0.73(−1.33, −0.14) *Traditional*; 8 years −0.19(−0.71, 0.33)15 years −0.41(−0.77, −0.04)). The *Ready-to-eat* trajectory had no association with IQ at either age (8 years 0.32(−4.31, 4.95), 15 years 1.11(−3.10, 5.33).

**Conclusions:**

The *Discretionary* and *Traditional* dietary pattern trajectories from 6 to 24 months of age, over the period when food patterns begin to emerge, are weakly associated with IQ in adolescence.

## Introduction

For at least 80 years, the concept that early life diet may have a lasting effect on cognitive outcomes has interested researchers[1] and appealed to the wider public. There is evidence from randomised controlled trials that diet in early life may influence cognitive development in childhood[2], [3]. For example, exclusive breastfeeding in the first 3 months of life is associated with 5.9-point higher intelligence quotient (IQ) score at 6 years of age (95% CI −1.0 to 12.8)[2]. While the mechanisms underpinning effects such as these are not understood, one hypothesis is that dietary factors may influence cognitive ability via changes to neural structure, tissue composition or epigenetic mechanisms[4], [5].

Since neural tissues undergo rapid development during the first few years of life, it is biologically plausible that this early developmental period may be sensitive to dietary factors. Only two studies have examined whether dietary patterns in infancy are associated with later cognitive ability[6], [7]. In a study involving n = 241 children, Gale and co-workers (2009) demonstrated that a standard deviation increase in ‘infant guidelines’ dietary pattern score at either 6 or 12 months was associated with around 2 to 3 point higher full-scale IQ scores at 4 years of age[8]. We have also shown that healthier dietary patterns are associated with IQ in a large sample (n = 7097)[7]. However, cross-sectional analyses such as these are problematic because they do not capture the rapid development of dietary patterns as infants make the transition from a milk-based diet to family foods, nor the correlation in diet measured at different times. For example, the infant who is breastfed may also be fed a healthier diet throughout childhood. One strategy for addressing the correlation between multiple measures of diet is to include past diet as a potential confounder in the analysis[9]. However, this method attempts to isolate the independent effect of diet at a particular time period rather than quantifying changes in diet over the total time period. Additionally, this method may introduce collinearity and not fully account for the correlations between multiple measures of diet. There are very few examples in the literature of how to model diet longitudinally[10], [11], [12], and to the best of our knowledge there are no examples of longitudinal modelling of dietary patterns as they emerge from infancy.

In this manuscript, we take a novel approach to longitudinal modelling of dietary patterns over the first two years of life. We examine dietary patterns in a UK population-based birth cohort at ages 6, 15 and 24 months and assign each pattern to a trajectory. We then examine the association between these dietary pattern trajectories and IQ assessed at 8 and 15 years of age. Analysis of IQ at both ages allows us to examine whether any effect present at 8 years persists into adolescence, when IQ is more indicative of adult IQ[13].

## Methods

### Ethics statement

Ethical approval for the study was obtained from the Avon Longitudinal Study of Parents and Children (ALSPAC) Law and Ethics Committee, and local Research Ethics Committees including; the Bristol and Weston Health Authority, the Southmead Health Authority, the Frenchay Health Authority, and the Central and South Bristol Research Ethics Committee. Written consent for participation in the ALSPAC study was provided by the child's parent or caregiver. The ALSPAC data was supplied to the authors in anonymized form.

### ALSPAC cohort

ALSPAC is a longitudinal, population-based birth cohort that was designed to investigate the determinants of health and development. Pregnant women who were expected to deliver between April 1991 and December 1992, and resided in the former county of Avon, UK were invited to participate. Of the 14541 women enrolled to the ALSPAC study, all but 69 had a known birth outcome and 14062 were live births. Data from (n = 10) triplet and quadruplet births have been removed from the dataset for confidentiality. The 13978 infants who survived to 1 year of age were eligible for the current study. ALSPAC data is made available to bona fide researchers by completing a proposal form available at the study website and submitting to the ALSPAC Executive Committee for approval (http://www.bristol.ac.uk/alspac/sci-com/). The authors of the present manuscript cannot share data directly with other researchers.

Diet data was collected by food frequency questionnaires sent to the primary caregiver when the study child was 6, 15 and 24 months of age. Questionnaires are available from the ALSPAC website (http://www.bristol.ac.uk/alspac/sci-com/quests/). As the diets of children develop between 6 and 24 months, the number and types of foods and beverages included in the questionnaire increased with age. The foods and beverages included in the questionnaires were determined by an experienced nutritionist (PE) and included 43 items at 6 months, 70 at 15 months and 72 at 24 months. Questionnaires included items on breastfeeding, formula feeding, and whether infants were fed commercially made (‘Ready-prepared’) baby foods and home-cooked foods. Caregivers were asked to report how many times per week a child currently consumed a food or beverage item. Children who had never been fed an item were assigned an intake of zero. No information on portion size was obtained. The questionnaires have not been validated against other diet assessments, but a comparison against diet diaries collected from a subset of n = 852 infants at 4 months is consistent with data collected by the corresponding questionnaire[14].

IQ was measured using an adapted form of the Wechsler Intelligence Scale for Children (WISC) Version III[15] when children were aged 8 years and the Wechsler Abbreviated Scale of Intelligence (WASI) at 15 years of age[16]. The IQ assessments were one of several tests conducted by trained psychologists at ALSPAC research clinics. At 8 years of age, alternate items from the WISC verbal and performance subtests were administered to ameliorate respondent fatigue, except for the coding subtest which was administered in full. Scores on each WISC subtest were calculated in a standard manner by summing individual items and multiplying by 2 for picture completion, information, arithmetic, vocabulary, comprehension and picture arrangement, multiplying by 1.67 for similarities and multiplying by 1.5 for object assembly and block design. Raw scores were converted to age-scaled scores and combined to determine Full Scale IQ (FSIQ) and subscales of Verbal (VIQ) and Performance IQ (PIQ). Due to limitations in clinic time, only two of the four WASI subtests (the Vocabulary and Matrix Reasoning subtests) were administered to study participants at 15 years of age, which provides an indication of general cognitive functioning.

Potential confounding factors included; maternal age at birth of the study child, maternal education, social class, marital status, maternal tobacco smoking during pregnancy, parity, family income, ethnicity, the number of children (<16 years old) living in the family home, stimulation in the home environment and duration of breastfeeding. These data were collected by postal questionnaires sent to the mother between 8 and 32 weeks gestation, unless described otherwise below. Maternal education was reported as the highest completed level on five categories from Certificate of Secondary Education (CSE), Vocational training, O(ordinary)-levels, A(advanced)-levels and degree or higher. Social class was categorised according to maternal occupation using standard UK classifications of occupation, ranging from class I (highest), II, III-non-manual, III-manual, IV, and V (lowest)[17]. Marital status was dichotomised into two categories; whether the mother had or did not have a partner. Tobacco smoking was divided into three categories; never smoked, has quit smoking or smoked in the last 2 months of pregnancy. Weekly family income was collected at 33 months postnatal as there was no measure of income at earlier ages and was divided into the following categories: £<200, £200–299, £300–399 and £>400 per week. The number of children living in the family home was collected at 6 months postnatal and was categorised as none, 1 and ≥2. Stimulation in the home environment was measured by an adaptation of the HOME questionnaire at 18 months of age[18]. Detailed breastfeeding data was collected prospectively by questionnaire sent at 4 weeks, 6, 15 and 24 months of age. Breastfeeding duration was divided into the following 5 categories; never breastfed, <1 month, 1 to <3 months, 3 to <6 months and ≥6 months. Further potential confounders collected by ALSPAC staff at delivery, from medical records or from birth notification included; sex, gestational age at birth, birth weight and singleton/multiple birth information. In addition to the confounding variables, maternal IQ was collected opportunistically from mothers who attended the clinic assessments when their child was 15 years old. Although maternal IQ was measured after collection of the child's dietary data (the exposure), we have treated it as a potential confounder because IQ is stable over adulthood[13] and has been identified as an important confounder in the association between breastfeeding and IQ[19]. The assessment of maternal IQ was conducted when the child was undergoing other clinic assessments and only when time was available (child assessments always took priority). Maternal IQ (n = 2907) was collected using the WASI as described above for the study child. Mothers who had IQ measured were not present at their child's WASI assessment. All of the potentially confounding factors described above were identified *a priori* by the authors. In response to peer-review, pre-pregnancy BMI and maternal alcohol consumption during pregnancy were included *a posteriori* as potentially confounding factors. Maternal alcohol was self-reported at 32 weeks gestation as the total number of drinks containing alcohol currently consumed per week, and was categorised into none, 1–7 drinks per week and >7 drinks per week. Pre-pregnancy BMI was collected during pregnancy from maternal report of pre-pregnancy weight (in kg) and height (m).

### Statistical analysis

Dietary trajectories were empirically determined by mapping the dietary patterns that were extracted by principal component analysis (PCA) at 6, 15 and 24 months of age. PCA is a multivariable technique that utilises correlations between foods to identify latent dietary patterns. We have previously described dietary patterns in the ALSPAC cohort at 6 (n = 7052), 15 (n = 5610) and 24 (n = 6366) months of age using PASW software (version 17.0 (formerly SPSS))[7], [20]. A dietary pattern score is calculated for each individual as a function of the contribution (the ‘loading’) that each food makes to the pattern, and the frequency that each food is consumed. Pattern scores are continuous, approximately normally distributed, have a mean equal to zero and standard deviation equal to one. The number of patterns extracted was determined by a break in the Scree plot, interpretability and consistency across ages. Four dietary patterns were extracted at each age. The patterns were examined for their similarity in the types of foods and their loadings to determine whether they would be suitable to be modelled as a trajectory. The mapping of each pattern to a trajectory was subjective and based on the foods with high loadings on each pattern. Each trajectory contained values from one pattern extracted at 6, 15 and 24 months of age. Trajectories were computed using multi-level Mixed Models in STATA (IC version 11.0). For multi-level modelling, the predictor variables were age (6, 15 and 24 months) and the outcome variable was the individual's dietary pattern score at each age. The dietary pattern scores contain repeated measures for each participant and are therefore not independent observations. Multi-level mixed models take into account the clustering of individuals' (repeated) dietary pattern scores. Both the intercept and slope were computed as a random effect, which allows the intercept to vary for each participant (i.e. reflecting different starting points on the trajectory) and the slopes to vary (reflecting different rates of change in pattern scores over time). The estimates of the different intercepts and slopes were then used as predictor variables in the analysis described below. The naming of the trajectories was based on the constituent food patterns (healthy, discretionary, traditional and ready-to-eat), but was nevertheless subjective and used to enhance the presentation of results and discussion.

Generalized linear models (GLM) with maximum likelihood estimation were used to examine the association between dietary pattern trajectories (predictors) and IQ scores at 8 years and 15 years (outcomes). The maximum likelihood method was used to reduce bias in estimating the within-subject effect[21], [22]. Regression models were adjusted firstly for other dietary pattern trajectories, then for the potentially confounding perinatal and sociodemographic variables described above. The regression coefficient for the slope of the dietary pattern trajectory was scaled to reflect a change in diet over the 18-month period between 6 to 24 months of age. Regression coefficients (β) and 95% confidence intervals were used to evaluate the strength and precision of the associations between dietary pattern trajectories and IQ.

Analyses were conducted on participants who had at least one WISC subscale measured at 8 years and/or one WASI subtest at 15 years (n = 7652). To minimise non-response bias, missing data on PCA pattern scores, covariables, WISC and WASI were imputed prior to calculating dietary pattern trajectories. The imputation was undertaken using Multiple Imputation for Chained Equations in STATA, as described by Royston *et al* [23]. Twenty imputed datasets were generated using 50 cycles of regression switching. The imputation model included IQ at 8 and 15 years, dietary pattern scores at all ages, all covariables and other additional variables that were not included in the fully-adjusted analytical model. The additional variables included: dietary pattern scores at age 3[24], Wechsler Preschool and Primary Scales of Intelligence collected from a 10% subset of the cohort at 4 years of age and scores on the Crown Crisp Experiential Index for anxiety and Edinburgh Postnatal Depression Scores at 8 months. The distribution of variables in the imputed analysis was similar to the complete case data (Table S1 in File S1). The multi-level mixed effect models used to create the dietary pattern trajectories and the analysis by GLM was undertaken for each of the imputed datasets separately and the findings were combined using Rubin's rules[25]. The results of imputed analyses are presented as the primary analyses and for completeness, the complete case analyses are reported in supplementary material (File S1).

## Results

Of the 13978 children in the core ALSPAC cohort who survived to 1 year of age, 6326 did not have IQ measured at either 8 and 15 years of age, 2619 had one or both WISC subscales measured only at 8 years, 555 had WASI measured only at 16 years, and 4478 had IQ measured at both 8 and 16 years of age. Characteristics of children who had IQ measured at least once differed from those who did not have IQ measured (Table 1). In particular, the mothers of children who had IQ measured were less likely to have smoked during pregnancy, a higher proportion were married, had completed O-level education or higher and were more likely to breastfeed their infant compared with children who did not have IQ measured.

**Table 1:** Comparison of characteristics between participants with IQ measured at 8 or 15 years and those that did not have IQ measured^1^.

	IQ measured at 8 or 15 years (n = 7652)			IQ not measured (n = 6326)			Mean difference (95% CI), [P value]^2^
	n with data		Mean (SD) or n (%)	n with data		Mean (SD) or n (%)	
**Birth and perinatal characteristics**							
Gestational age at birth (wk)	7652		39.4±1.9	6326		39.4±1.9	−0.03 (−0.1, 0.03) [0.30]
Birth weight (kg)	7562		3.41±0.55	6326		3.37±0.57	−0.04 (−0.06, −0.03) [<0.001]
Birth length (cm)	6049		50.6±2.5	4487		50.5±2.5	−0.1 (−0.2, −0.0) [0.01]
Sex (Male)	7652		3799 (50)	6324		3421 (54)	[<0.001]
Singleton	7652		7442 (97)	6326		6175 (98)	[0.19]
Maternal age at birth (y)	7652		29.0±4.6	6326		26.8±5.1	−2.2 (−2.4, −2.1) [<0.001]
Parity	7370			5559			[<0.001]
0			3423 (46)			2349 (42)	
1			2633 (36)			1906 (34)	
2			974 (13)			877 (16)	
3			263 (4)			277 (5)	
≥4			77 (1)			150 (3)	
Alcohol intake at 32 wk gestation	4243			2648			[<0.001]
None			2804 (66)			1896 (72)	
1–7 per week			1179 (28)			588 (22)	
>7 per week			260 (6)			164 (6)	
Maternal pre-pregnancy BMI	6875		22.9±3.7	4659		22.9±4.0	−0.01 (−0.2, 0.1) [0.89]
**Maternal and family characteristics**							
Tobacco smoking		7079		4355			[<0.001]
Never			3918 (55)		1886 (43)		
Quit			2177 (31)		1280 (29)		
Smoked during last trimester of pregnancy			984 (14)		1189 (27)		
Any breastfeeding		7008		4133			[<0.001]
Never			1318 (19)		1438 (35)		
≤1 month			1104 (16)		706 (17)		
1 to <3 months			1119 (16)		648 (16)		
3 to <6 months			992 (14)		435 (11)		
≥6 months			2475 (35)		906 (22)		
White ethnicity		7616	7277 (96)	6058	5629 (93)		[<0.001]
Maternal marital status		7460		5627			[<0.001]
First marriage			5556 (75)		3403 (61)		
Subsequent marriage/s			478 (6)		369 (7)		
Widowed/divorced/separated			364 (5)		418 (7)		
Never married			1062 (14)		1437 (26)		
Maternal education^3^		7356		5062			[<0.001]
None/CSE			1000 (14)		1504 (30)		
Vocation			651 (9)		573 (11)		
O level			2590 (35)		1706 (34)		
A level			1953 (27)		841 (17)		
Degree or higher			1163 (16)		438 (9)		
Maternal social class^4^		6353		3710			[<0.001]
I			432 (7)		159 (4)		
II			2205 (35)		964 (26)		
II (non-manual)			2673 (42)		1631 (44)		
III (manual)			430 (7)		359 (10)		
IV			523 (8)		469 (13)		
V			90 (1)		128 (3)		
Family income (£ per week)		6088		2753			[<0.001]
<100			393 (6)		377 (14)		
100–199			946 (16)		615 (22)		
200–299			1757 (29)		756 (27)		
300–399			1383 (23)		496 (18)		
≥400			1609 (26)		509 (18)		
HOME score^5^		7165	8.2±2.2	4307	7.8±2.3		−0.3 (−0.4, −0.3) [<0.001]
Number of other children		7112		4274			[<0.001]
0			3212 (45)		1713 (40)		
1			2630 (37)		1571 (37)		
2			977 (14)		698 (16)		
3			232 (3)		208 (5)		
≥4 or more			61 (1)		84 (1)		

1 Abbreviations; BMI, body mass index; CSE, Certificate of Secondary Education; IQ, intelligence quotient; WASI, Wechsler Abbreviated Scale of Intelligence; WISC, Wechsler Intelligence Scale for children

2 Statistical tests were used to compare characteristics of participants who had IQ measured at 8 or 15 years, against participants who had no IQ measurements. Continuous variables were compared by using independent t-test and categorical variables were compared by using χ^2^ tests.

3 Maternal education is reported as the highest completed level on five ordinal categories from Certificate of Secondary Education(CSE), Vocational training, O(ordinary)-level (taken by the top 25% of CSE at 15 years), A(advanced)-level (involving 2 years of study beyond O-level) and degree or higher. The CSE, O-levels and A-levels are completed at secondary school.

4 Social class was categorized according to maternal occupation during pregnancy, according to standard UK classifications of occupation, ranging from class I (highest), II, III-non-manual, III-manual, IV, and V (lowest)[17].

5 Stimulation in the home environment was measured by an adaptation of the HOME questionnaire at 18 months of age[18].

### Mapping of dietary pattern trajectories

**Figure 1:**
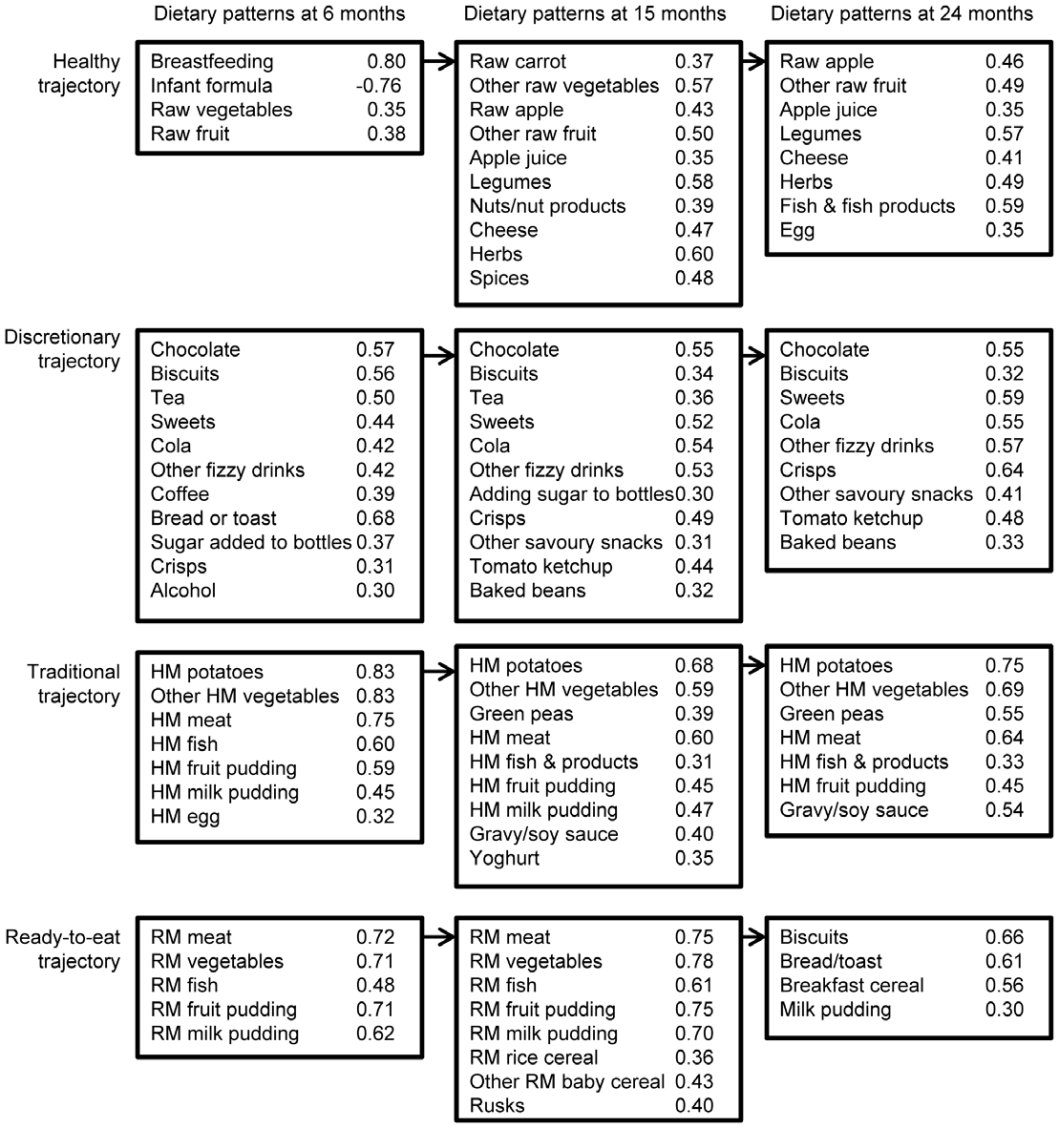
Mapping of dietary patterns at age 6, 15 and 24 months to trajectories. Only foods with loadings ≥±0.3 are given in the figure. A full list of foods and their loadings are provided in references [7], [20]. Abbreviations; HM, home-made; RM, ready-prepared infant/toddler foods.

Figure 1 shows the dietary patterns extracted at 6, 15 and 24 months of age and the foods with loadings >0.3. Each of these patterns was used to model the four dietary pattern trajectories. Assignment to a trajectory was according to the type of foods that loaded most strongly on each dietary pattern. For example, a traditional British style pattern (characterised by meat, cooked vegetables and puddings) was identified at all ages and scores on each of these patterns were connected to model the ‘*Traditional*’ dietary pattern trajectory. Similarly a dietary pattern characterised by discretionary foods such as crisps and chocolate were identified at all ages and these patterns were used to model the ‘*Discretionary*’ trajectory. The ‘*Healthy*’ trajectory brought together the *Breastfeeding* pattern at 6 months and *Contemporary* patterns at 15 and 24 months. Finally, the *Ready-prepared baby foods* patterns at 6 and 15 months (characterised by foods that are purchased in jars, tins or packets) and the *Ready-to-eat* pattern at 24 months were used to generate the trajectory named ‘*Ready-to-eat*’. The common construct underlying the *Ready-to-eat* trajectory was that the foods loading on the trajectory required little preparation or cooking. The intercept and slope for each of the trajectories obtained by multi-level Mixed Models was approximately normally distributed. The range of values for the trajectory slopes are as follows: *Healthy* −0.15, 0.24; *Discretionary* −0.16, 0.36; *Traditional* −0.12, 0.22, and *Ready-to-eat* −0.08, 0.04.

### Associations between dietary pattern trajectories and IQ at 8 and 15 years of age

The fully-adjusted association between the dietary pattern trajectories and IQ at 8 and 15 years of age are shown in Table 2. The sequential modelling of each trajectory's intercept and slope in a bivariate analysis (model 1), after adjustment for other trajectories (model 2), perinatal covariables (model 3) and the final fully-adjusted model that also includes all sociodemographic variables (model 4) for FSIQ, VIQ and PIQ at 8 are shown in the supplementary tables provided in the online supporting information (Table S2, S3 and S4 in File S1, respectively) and FSIQ at 15 years of age (Table S5 in File S1). These online tables show that association between dietary pattern trajectory and IQ is attenuated by adjustment for potentially confounding variables, as indicated by the lowering of beta-coefficients and narrowing of confidence intervals after adjustment. For example, the β coefficient (95% CI) for a 1 SD increase in the slope of the *Healthy* dietary pattern trajectory and IQ at 8 years is 5.22 (4.37, 6.07) in unadjusted analyses, 3.88 (2.98, 4.78) with adjustment for other dietary pattern trajectories, 2.87 (1.94, 3.79) when perinatal variables are included and 1.07 (0.17, 1.97) when including sociodemographic variables (i.e. the final, fully adjusted model).

**Table 2:** Fully adjusted associations between dietary pattern trajectories from (6 to 24 months) and IQ^1^ at 8 and 15 years of age (n = 7652)^2^.

		8 years									16 years		
		FSIQ			VIQ			PIQ			FSIQ		
Trajectory		β^3^	95% CI	P	β	95% CI	P	β	95% CI	P	β	95% CI	P
Healthy	Intercept	0.66	−0.20, 1.51	0.134	1.00	0.08, 1.92	0.033	0.03	−0.86, 0.93	0.939	0.75	0.00, 1.50	0.049
Healthy	Slope	1.07	0.17, 1.97	0.020	0.95	0.02, 1.88	0.046	1.03	−0.01, 2.06	0.051	0.49	−0.28, 1.26	0.208
Discretionary	Intercept	−0.92	−1.98, 0.14	0.089	−1.51	−2.56, −0.45	0.006	0.03	−1.20, 1.26	0.959	−0.29	−1.14, 0.56	0.503
Discretionary	Slope	−0.35	−1.03, 0.33	0.307	−0.52	−1.16, 0.12	0.110	−0.10	−0.94, 0.73	0.802	−0.73	−1.33, −0.14	0.017
Traditional	Intercept	1.03	−0.20, 2.26	0.100	0.97	−0.46, 2.19	0.196	0.98	−0.26, 2.23	0.121	0.09	−0.92, 1.09	0.860
Traditional	Slope	−0.19	−0.71, 0.33	0.466	−0.11	−0.64, 0.41	0.666	−0.20	−0.79, 0.39	0.495	−0.41	−0.77, −0.04	0.031
Ready-to-eat	Intercept	−3.83	−9.76, 2.11	0.205	−2.61	−9.38, 4.15	0.444	−4.42	−12.21, 3.38	0.262	−0.14	−5.87, 5.60	0.962
Ready-to-eat	Slope	0.32	−4.31, 4.95	0.891	2.42	−3.51, 8.35	0.414	−2.52	−8.53, 3.50	0.404	1.11	−3.10, 5.33	0.597

1 Abbreviations: FSIQ, full scale intelligence quotient; IQ, intelligence quotient; PIQ, performance intelligence quotient; VIQ, verbal intelligence quotient

2 Eligible participants were children who had at least one measurement of IQ collected at either 8 or 15 years of age (n = 7652). IQ was measured using the Wechsler Intelligence Scale for Children at 8 years and the Wechsler Abbreviated Scale of Intelligence at 15 years of age. Incomplete IQ or missing covariable data was imputed by Multiple Imputation.

3 Analyses show the beta-coefficient from multivariable linear regression analysis using generalized linear models and adjusted for the following covariables; sex, gestational age at birth, birth weight, ethnicity, singleton/twin, maternal age, parity, social class (according to standard UK classifications of occupation at the time of birth[17]), maternal education, other children, family income, maternal smoking, stimulation in the home environment (using an adaptation of the HOME questionnaire[18]) and maternal IQ (measured by WASI when the study child was 15 years of age). A one standard deviation change in *Healthy* dietary pattern trajectory from 6 to 24 months of age is associated with 0.66 (95% CI −0.20, 1.51) higher FSIQ scores at 8 years of age.

While an increasing slope on the *Healthy* dietary pattern trajectory is associated with approximately 1-point higher FSIQ scores at 8 years of age, the intercept and slope of the *Discretionary* and *Traditional* trajectories are less convincing (Table 2). Compared with the other trajectories, the *Read-to-eat* trajectory has wide confidence intervals around the intercept and slope, and there appears to be no clear association with IQ at 8 years of age. The associations between dietary pattern trajectories and VIQ or PIQ at 8 years of age are generally consistent in the direction and magnitude as for FSIQ.

At 15 years of age, the association between dietary pattern trajectories from 6 to 24 months of age and IQ are similar to the findings at 8 years although the size of the effect differs. The slope of the *Healthy* pattern trajectory is positively associated with IQ ((0.49 (but note the confidence intervals are −0.28 to 1.26), whereas the slopes of the *Discretionary* and *Traditional* trajectories are clearly negative ((−0.73 (−1.33, −0.14) and −0.41 (−0.77, −0.04), respectively). Consistent with IQ at 8 years of age, the confidence intervals around the *Ready-to-eat* trajectory are wide and there is no clear association with IQ at 15 years of age (1.11 (−3.10, 5.33)).

Compared with the results of the imputed analyses presented in Table 2, there is less precision in the complete-case analyses (Tables S6 to S9 in File S1) because the confidence intervals are wider and there are some inconsistencies across analyses. For example, the complete-case fully-adjusted model of the association between the *Discretionary* pattern trajectory and FSIQ at 8 years is −1.51 (−5.99, 2.97) for the intercept and 0.10 (−2.08, 2.29) for the slope, compared with the imputed analysis (−0.92 (−1.98, 0.14) and −0.35 (−1.03, 0.33), respectively).

## Discussion

We modelled a longitudinal measure of the whole diet as it emerges from infancy through the transition to solid foods and have shown that some of the dietary trajectories are weakly associated with IQ at 8 and 15 years of age. Given that the majority of previous studies on infant feeding and IQ have focussed on breastfeeding, our study additionally highlights the contribution of foods fed to infants and toddlers. The direction of association between dietary pattern trajectories and IQ was similar at 8 and 15 years, which supports the concept that the effect of dietary patterns in early life may persist over time. The positive association between the *Healthy* trajectory and IQ at age 8 was weakened by 15 years, whereas the negative associations with the *Discretionary* and *Traditional* trajectories were more pronounced by 15 years. The *Ready-to-eat* trajectory had wide confidence intervals and our *post hoc* interpretation of the data raises doubts over whether *Ready-to-eat* truly reflects a dietary pattern trajectory. The foods that contributed to the *Ready-to-eat* trajectory at 6 and 15 months included ready-prepared baby foods (such as rice cereal, baby foods in tins, jars and packets), but quite different foods loaded on the *Ready to eat* trajectory at 24 months (such as bread, breakfast cereals, biscuits and milk pudding). *A priori* we assumed that the change in foods consumed at 15 and 24 months reflected a shift from conveniently-prepared baby foods towards family foods that required little preparation or cooking. It is possible that the baby food patterns at 6 and 15 months reflect a transient eating pattern that is different from the Ready-to-eat pattern at 24 months of age and we now have less confidence in the existence of a *Ready-to-eat* trajectory.

By using similar dietary patterns over time to model trajectories, we have been able to examine the whole diet in a longitudinal manner. This is an advance in nutritional epidemiology when compared with cross-sectional analyses or when past diet is used as a confounder in the analysis of current diet. The intercepts and slopes were used as continuous variables and therefore we were able to exploit their variability and the full range of values to examine the association with IQ. In addition to being more parsimonious, the modelling used in the present study makes the most of the repeated measures of diet collected from cohort studies. The timing of the dietary questionnaires coincides with important stages of an infants' diet; the 6-month questionnaire captures the first exposures to food, the 15-month questionnaire is at a time when a toddler becomes an accomplished eater of a variety of foods and by 24 months of age, the transition to the family diet is usually complete. Different questionnaires were used to measure diet at each age, which is necessary because of the smaller variety and the type of foods fed to infants (e.g. teething rusks, rice cereal, infant formula) compared with the larger variety and types of foods fed to toddlers (e.g. bread, cow's milk). This adds some complexity to the analysis because the pattern scores at each age are a function of the contribution that each food makes to the pattern and the frequency at which the food is consumed. Hence the trajectories are based on the assumption that changes in pattern scores reflect a true change in a latent dietary construct and are not a result of different factor loadings at each age. We had a large sample size (n = 7652) and addressed non-response bias by using multiple imputation. However it is not possible to know whether the effects are consistent among the group of children who did not have IQ measured. Attrition bias is a problem common to all cohort studies however research from Norway that compares population-based studies with cohort studies indicate that this may be less of a problem than previously thought[26]. While we adjusted for a wide range of confounders, it is not possible to rule out residual or unmeasured confounding. The role of unmeasured confounding may be especially important given the small effects of diet observed in this study. We were unable to test whether the association between each trajectory and IQ was due to total dietary energy intake because we did not have a concurrent measure of dietary energy from 6–24 months of age. The associations between dietary patterns and total dietary energy are weak, they differ over time and across patterns[27], and because children from the ALSPAC cohort are generally well-nourished it is unlikely that the association between the trajectories and IQ is due to energy intake. Since nutrient intakes cannot be derived from the dietary trajectories we can only speculate about the potential mechanism. We hypothesise that the compared with the *Discretionary* trajectory, the *Healthy* trajectory may have a higher density of nutrients that have been suggested as important for cognitive development (such as iron, zinc and/or B vitamins)[28], [29], and this is probably due to higher intakes of fruit, vegetables, dairy and fish[30]. Overall, dietary pattern trajectories are an advancement in the longitudinal modelling of diet, but a caveat is that it would only be possible to generate a trajectory if the dietary patterns are similar and stable over time; any transient or newly-emerging patterns could not be modelled as a trajectory[31].

Methods for modelling dietary patterns measured on at least three occasions have been published in studies that involve older children or adults, and all involve associations with body mass index rather than IQ. For example, Mikkila *et al* conducted longitudinal modelling of 2 dietary patterns measured on 3 occasions over a 21-year period using data from the Cardiovascular Risk in Young Finns Study[10]. The participants were aged 3 to 18 years at study entry (n∼1200). A mixed linear regression model with repeated measurements showed that the *Traditional* (Finnish) pattern trajectory was associated with poorer cardiovascular risk, while the *Healthy* pattern trajectory was associated with better cardiovascular risk factors such as serum cholesterol, blood pressure and BMI. This elegant modelling is unable to be applied in our research as IQ cannot be measured in infancy (when the diet data was collected). By comparison, Mishra and co-workers (2006) dichotomised food intake data into consumers or non-consumers, conducted a factor analysis for binary data, summed food items that loaded on each pattern for the three separate occasions and then used mixed models to examine the association between pattern score trajectories and BMI[11]. Although the procedure for obtaining a pattern score is simplified, this methodology does not take into account the extent to which a food contributes to a pattern (i.e. the loading) or the frequency by which foods are consumed. Finally, Pachuki analysed dietary data from the Offspring Cohort of the Framingham Heart Study on three occasions using cluster analysis, they ordered the clusters according to quality using an index, and then used the probability of moving to a higher or lower quality cluster as a predictor of BMI[12]. This method is complex to interpret but it shows that movement to clusters with poorer diet quality was associated with higher BMI.

**Figure 2:**
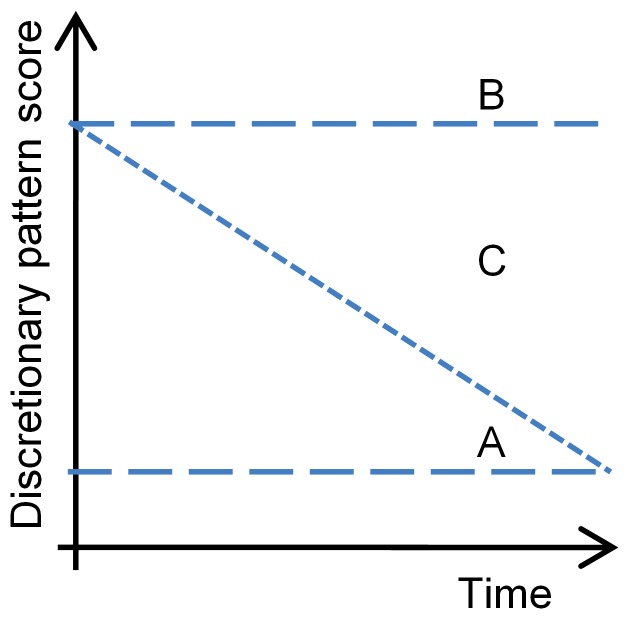
Examples of *Discretionary* pattern trajectories over time. Infant ‘A’ scores low on the *Discretionary* pattern at 6 months (low intercept) and continues to score low on the *Discretionary* pattern (level trajectory). Infant ‘B’ scores high on the *Discretionary* pattern at 6 months (high intercept) and continues with a high score (level trajectory). Infant ‘C’ could be the subject of interventions that target children with high scores and aim to reduce scores in the *Discretionary* trajectory.

Understanding the extent to which *changes* in diet over time influences IQ has important public health implications. The finding that dietary pattern trajectories are associated with IQ suggests that it may be possible to improve IQ outcomes for young children by improving their diet. As shown in Figure 2, we posit that the ideal dietary pattern trajectory is shown by infant ‘A’ who scores low on the *Discretionary* pattern at 6 months (low intercept) and continues to score low on the *Discretionary* pattern to 24 months of age (level trajectory). However it is infant ‘B’, who scores high on the *Discretionary* pattern at 6 months (high intercept) and continues with high scores (level trajectory), should be the target of public health nutrition interventions. Using data from the present study, we estimated that an intervention to shift all infants whose diets begin in the highest tertile of *Discretionary* pattern scores at 6 months to a lower (healthier) dietary trajectory by 24 months of age (infant ‘C’) would be associated with a 0.52 -point higher in IQ at 8 years (95% CI −0.61, 1.65) and 1.16 point at 15 years (95%CI 0.10, 2.23). In terms of interventions to improve IQ, this is a relatively small effect of diet. By comparison, an intervention designed to prolong exclusive breastfeeding is associated with a 5.9-point higher IQ at 6 years of age[2]. A comparable effect size in this research is comparing mothers who have a degree with mothers who have CSE or vocational qualifications (Table S2 in File S1). The small effect size of early diet on adolescent IQ will raise concern over whether scarce resources should be invested in improving early life diet solely on the basis of enhancing IQ. However, economic modelling suggests that even small incremental gains in IQ at a population level may have measurable effects on the development of human capital[32].

### Conclusion

In this study, we have utilised data from a prospective birth cohort, applied methods for measuring the whole diet (rather than individual nutrients) longitudinally, taken care to minimise non-response bias and accounted for a wide range of potential confounders We find that the *Healthy* dietary pattern trajectory is associated with IQ at 8 and the *Discretionary* and *Traditional* trajectories are associated with IQ at 15 years. This suggests that diet from 6 to 24 months of age, when neural tissues are undergoing rapid development, has a small but persistent association with IQ.

## Supporting Information

File S1 **File S1 contains the following tables.** Table S1: Comparison of distributions of diet pattern (exposure), IQ (outcome) and covariables for observed and imputed datasets. Table S2: Associations between dietary pattern trajectories measured from 6 to 24 months and full scale IQ at 8 years of age (n = 7652). Table S3: Associations between dietary pattern trajectories measured from 6 to 24 months and verbal IQ at 8 years of age (n = 7652). Table S4: Associations between dietary pattern trajectories measured from 6 to 24 months and performance IQ at 8 years of age (n = 7652). Table S5: Associations between dietary pattern trajectories measured from 6 to 24 months and full scale IQ at 15 years of age (n = 7652). Table S6: Complete case analysis of the associations between dietary pattern trajectories measured from 6 to 24 months and full scale IQ at 8 years of age (n = 950). Table S7: Complete case analysis of the associations between dietary pattern trajectories measured from 6 to 24 months and verbal IQ at 8 years of age (n = 954). Table S8: Complete case analysis of the associations between dietary pattern trajectories measured from 6 to 24 months and performance IQ at 8 years of age (n = 952). Table S9: Complete case analysis of the associations between dietary pattern trajectories measured from 6 to 24 months and full scale IQ at 15 years of age (n = 956).
(DOCX)
