# Supplementary material for: Do Dietary Trajectories between Infancy and Toddlerhood Influence IQ in Childhood and Adolescence? Results from a Prospective Birth Cohort Study
Source: PLoS One. 2013 Mar 13;8(3):e58904. doi: 10.1371/journal.pone.0058904 (PMC3596350; doi:10.1371/journal.pone.0058904)
Supplement: File S1 — contains the following tables. Table S1: Comparison of distributions of diet pattern (exposure), IQ (outcome) and covariables for observed and imputed datasets. Table S2: Associations between dietary pattern trajectories measured from 6 to 24 months and full scale IQ at 8 years of age (n = 7652). Table S3: Associations between dietary pattern trajectories measured from 6 to 24 months and verbal IQ at 8 years of age (n = 7652). Table S4: Associations between dietary pattern trajectories measured from 6 to 24 months and performance IQ at 8 years of age (n = 7652). Table S5: Associations between dietary pattern trajectories measured from 6 to 24 months and full scale IQ at 15 years of age (n = 7652). Table S6: Complete case analysis of the associations between dietary pattern trajectories measured from 6 to 24 months and full scale IQ at 8 years of age (n = 950). Table S7: Complete case analysis of the associations between dietary pattern trajectories measured from 6 to 24 months and verbal IQ at 8 years of age (n = 954). Table S8: Complete case analysis of the associations between dietary pattern trajectories measured from 6 to 24 months and performance IQ at 8 years of age (n = 952). Table S9: Complete case analysis of the associations between dietary pattern trajectories measured from 6 to 24 months and full scale IQ at 15 years of age (n = 956). (DOCX) [file pone.0058904.s001.docx]

**Supporting Information File S1**

**Table S1:** Comparison of distributions of diet pattern (exposure), IQ (outcome) and covariables for observed and imputed datasets

| Variable | Observed dataset | | Twenty imputed datasets^1^  (n=7652) |
| --- | --- | --- | --- |
|  | n | Distribution^2^ | Distribution^2^ |
| WPPSI^3^ at 4 years |  |  |  |
| Full scale IQ | 1016 | 104.4 ± 14.2 | 102.6 ± 8.5 |
| Verbal IQ | 1013 | 100.1 ± 13.6 | 99.1 ± 8.9 |
| Performance IQ | 1013 | 108.0 ± 14.6 | 106.0 ± 11.1 |
| WISC at 8 years |  |  |  |
| Full scale IQ | 7044 | 104.2 ± 16.5 | 103.6 ± 16.1 |
| Verbal IQ | 7074 | 107.2 ± 16.8 | 106.6 ± 16.5 |
| Performance IQ | 7067 | 99.6 ± 17.1 | 99.2 ± 16.8 |
| WASI at 15 years |  |  |  |
| Full scale IQ | 4720 | 92.0 ± 13.0 | 92.2 ±12.0 |
| Verbal IQ (t-score) | 5032 | 45.6 ± 11.8 | 45.5 ± 11.2 |
| Performance IQ (t-score) | 4721 | 43.7 ± 9.2 | 44.2 ± 8.6 |
| Dietary patterns at 6 months |  |  |  |
| 1. Home-prepared traditional | 4604 | 0.04 ± 0.99 | 0.02 ± 1.00 |
| 2. Discretionary | 4604 | -0.10 ± 0.78 | -0.05 ± 0.88 |
| 3. Ready-prepared baby foods | 4604 | -0.02 ± 0.97 | -0.01 ± 0.98 |
| 4. Breastfeeding | 4604 | 0.07 ± 1.03 | 0.06 ± 1.02 |
| Dietary patterns at 15 months |  |  |  |
| 1. Contemporary | 3725 | 0.05 ± 0.99 | 0.07 ± 1.02 |
| 2. Discretionary | 3725 | -0.07 ± 0.92 | -0.06 ± 0.97 |
| 3. Ready-prepared baby foods | 3725 | -0.04 ± 0.91 | -0.02 ± 0.96 |
| 4. Home-prepared traditional | 3725 | -0.03 ± 0.96 | -0.01 ± 0.99 |
| Dietary patterns at 24 months |  |  |  |
| 1. Home-prepared traditional | 4399 | 0.01 ± 1.00 | 0.00 ± 1.00 |
| 2. Contemporary | 4399 | 0.05 ± 1.00 | 0.01 ± 1.01 |
| 3. Discretionary | 4399 | -0.06 ± 0.95 | -0.02 ± 0.98 |
| 4. Ready-to-eat | 4399 | 0.09 ± 0.98 | 0.02 ± 1.00 |
| Gestational age at birth (wk) | 7652 | 39.4 ± 1.8 | 39.4 ± 1.8 |
| Birth weight (kg) | 7562 | 3.41 ± 0.55 | 3.41 ± 0.55 |
| Birth length (cm) | 6049 | 50.6 ± 2.5 | 50.6 ± 2.6 |
| Sex (Male) | 7652 | 3799 (50) | (50) |
| Maternal age at birth (y) | 7652 | 29.0 ± 4.6 | 29.0 ± 4.6 |
| Singleton | 7652 | 7442 (97) | (97) |
| Parity | 7370 |  |  |
| 0 |  | 3423 (46) | (46) |
| 1 |  | 2633 (36) | (36) |
| 2 |  | 974 (13) | (13) |
| 3 |  | 263 (4) | (4) |
| ≥4 |  | 77 (1) | (1) |
| Tobacco smoking | 7079 |  |  |
| Never |  | 3918 (55) | (54) |
| Quit |  | 2177 (31) | (31) |
| Smoked during pregnancy |  | 984 (14) | (15) |
| Any breastfeeding | 7008 |  |  |
| Never |  | 1318 (19) | (20) |
| ≤1 month |  | 1104 (16) | (16) |
| 1 to <3 months |  | 1119 (16) | (16) |
| 3 to <6 months |  | 992 (14) | (14) |
| ≥6 months |  | 2475 (35) | (34) |
| White ethnicity | 7616 | 7277 (96) | (96) |
| Maternal marital status | 7460 |  |  |
| First marriage |  | 5556 (75) | (74) |
| Subsequent marriage/s |  | 478 (6) | (6) |
| Widowed/divorced/separated |  | 364 (5) | (5) |
| Never married |  | 1062 (14) | (15) |
| Maternal education^4^ | 7356 |  |  |
| None/CSE |  | 1000 (14) | (14) |
| Vocation |  | 651 (9) | (9) |
| O level |  | 2590 (35) | (35) |
| A level |  | 1953 (27) | (26) |
| Degree or higher |  | 1163 (16) | (16) |
| Maternal social class^5^ | 6353 |  |  |
| I |  | 432 (7) | (6) |
| II |  | 2205 (35) | (33) |
| III (non-manual) |  | 2673 (42) | (42) |
| III (manual) |  | 430 (7) | (7) |
| IV |  | 523 (8) | (10) |
| V |  | 90 (1) | (2) |
| Family income (£ per week) | 6088 |  |  |
| <100 |  | 393 (6) | (7) |
| 100-199 |  | 946 (16) | (16) |
| 200-299 |  | 1757 (29) | (29) |
| 300-399 |  | 1383 (23) | (22) |
| ≥400 |  | 1609 (26) | (25) |
| HOME score^6^ | 7165 | 8.2 ± 2.2 | 8.1 ± 2.2 |
| Number of other children | 7112 |  |  |
| 0 |  | 3212 (45) | (45) |
| 1 |  | 2630 (37) | (37) |
| 2 |  | 977 (14) | (14) |
| 3 |  | 232 (3) | (3) |
| ≥4 |  | 61 (1) | (1) |
| Maternal IQ | 2907 | 99.0 ± 13.8 | 97.8 ± 13.5 |
| Alcohol intake during pregnancy | 4243 |  |  |
| None |  | 2804 (66) | (67) |
| 7 drinks per week |  | 1179 (28) | (27) |
| >7 drinks per week |  | 260 (6) | (6) |
| Maternal Pre-pregnancy BMI | 6875 | 22.9 ± 3.7 | 22.9 ± 3.7 |

^1^ The imputation was conducted under the missing at random assumption by chained equations in STATA (IC version 12.0)[1]. In addition to all IQ outcomes, diet pattern scores and covariables, additional variables that predicted missingness were included in the imputation model. These additional variables included: diet pattern scores at age 3[2], maternal scores on the Crown Crisp Experiential Index for anxiety and Edinburgh Postnatal Depression Scores at 6 months, and also Wechsler Preschool and Primary Scale of Intelligence scores which was collected from a 10% subset of the cohort at 4 years. The imputation was conducted on all participants followed by analyses restricted to participants who had at least one measurement of IQ at 8 or 15 years of age (n=7652).

^2^ Distributions for observed data are shown as mean ± SD for continuous variables and n (%) for categorical variables. For imputed datasets, the overall percent is shown rather than the n (%) for each of the 20 datasets.

^3^ Abbreviations: BMI, body mass index; CSE, Certificate of Secondary Education; IQ, intelligence quotient; WASI, Wechsler Abbreviated Scale of Intelligence; WISC, Wechsler Intelligence Scale for children; WPPSI, Wechsler Preschool and Primary Scale of Intelligence

^4^ Maternal education is reported as the highest completed level on five ordinal categories from Certificate of Secondary Education(CSE), Vocational training, O(ordinary)-level (taken by the top 25% of CSE at 15 years), A(advanced)-level (involving 2 years of study beyond O-level) and degree or higher. The CSE, O-levels and A-levels are completed at secondary school.

^5^ Social class was categorized according to maternal occupation during pregnancy, according to standard UK classifications of occupation, ranging from class I (highest), II, III-non-manual, III-manual, IV, and V (lowest)[3].

^6^ Stimulation in the home environment was measured by an adaptation of the HOME questionnaire at 18 months of age[4].

**Table S2:** Associations between dietary pattern trajectories measured from 6 to 24 months and full scale IQ^1^ at 8 years of age (n=7652)^1^

|  | Model 1^2^ | | | | Model 2 | | | | Model 3 | | | | Model 4 | | | |
| --- | --- | --- | --- | --- | --- | --- | --- | --- | --- | --- | --- | --- | --- | --- | --- | --- |
|  | β^3^ | 95% CI | | p | β^3^ | 95% CI | | p | β^3^ | 95% CI | | p | β^3^ | 95% CI | | p |
|  |  | Lower | Upper |  |  | Lower | Upper |  |  | Lower | Upper |  |  | Lower | Upper |  |
| Healthy Intercept | 6.09 | 5.43 | 6.75 | <0.001 | 4.84 | 4.16 | 5.52 | <0.001 | 2.48 | 1.64 | 3.33 | <0.001 | 0.66 | -0.20 | 1.51 | 0.134 |
| Healthy slope | 5.22 | 4.37 | 6.07 | <0.001 | 3.88 | 2.98 | 4.78 | <0.001 | 2.87 | 1.94 | 3.79 | <0.001 | 1.07 | 0.17 | 1.97 | 0.020 |
| Discretionary Intercept | -6.35 | -7.26 | -5.44 | <0.001 | -5.13 | -6.13 | -4.14 | <0.001 | -2.91 | -3.99 | -1.84 | <0.001 | -0.92 | -1.98 | 0.14 | 0.089 |
| Discretionary slope | -2.82 | -3.46 | -2.18 | <0.001 | -1.85 | -2.56 | -1.13 | <0.001 | -1.18 | -1.88 | -0.49 | 0.001 | -0.35 | -1.03 | 0.33 | 0.307 |
| Traditional Intercept | 3.15 | 1.94 | 4.37 | <0.001 | 2.44 | 1.20 | 3.69 | <0.001 | 1.87 | 0.61 | 3.12 | 0.004 | 1.03 | -0.20 | 2.26 | 0.100 |
| Traditional slope | -0.88 | -1.46 | -0.29 | 0.004 | -0.25 | -0.82 | 0.31 | 0.375 | -0.18 | -0.72 | 0.36 | 0.499 | -0.19 | -0.71 | 0.33 | 0.466 |
| Ready-to-eat Intercept | 16.34 | -1.25 | 33.93 | 0.067 | 9.72 | -0.99 | 20.44 | 0.074 | 4.60 | -3.90 | 13.12 | 0.280 | -3.83 | -9.76 | 2.11 | 0.205 |
| Ready-to-eat slope | 20.41 | 3.28 | 37.54 | 0.022 | 11.14 | 1.14 | 21.14 | 0.031 | 7.37 | -0.47 | 15.20 | 0.064 | 0.32 | -4.31 | 4.95 | 0.891 |
| Female |  |  |  |  |  |  |  |  | 0.08 | -0.61 | 0.78 | 0.818 | -0.03 | -0.70 | 0.64 | 0.937 |
| Gestational age at birth (wk) 37-42 |  |  |  |  |  |  |  |  | Referent | | | | Referent | | | |
| <33 |  |  |  |  |  |  |  |  | -2.22 | -5.91 | 1.45 | 0.236 | -2.19 | -5.75 | 1.36 | 0.227 |
| 33-36 |  |  |  |  |  |  |  |  | 1.94 | 0.18 | 3.70 | 0.031 | 1.91 | 0.18 | 3.64 | 0.030 |
| >42 |  |  |  |  |  |  |  |  | 0.53 | -4.6 | 2.66 | 0.840 | 1.05 | -3.93 | 6.04 | 0.679 |
| Birth weight (kg) |  |  |  |  |  |  |  |  | 2.81 | 2.05 | 3.57 | <0.001 | 2.23 | 1.49 | 2.97 | <0.001 |
| White Ethnicity |  |  |  |  |  |  |  |  | 1.81 | 0.08 | 3.54 | 0.040 | 0.91 | -0.78 | 2.60 | 0.292 |
| Twin |  |  |  |  |  |  |  |  | -2.46 | -4.68 | -0.25 | 0.030 | -2.47 | -4.76 | -0.17 | 0.036 |
| Maternal age (y) |  |  |  |  |  |  |  |  | 0.47 | 0.38 | 0.55 | <0.001 | 0.20 | 0.11 | 0.29 | <0.001 |
| Parity |  |  |  |  |  |  |  |  | -2.57 | -3.02 | -2.13 | <0.001 | -1.14 | -2.02 | -0.27 | 0.010 |
| Breastfed (never) |  |  |  |  |  |  |  |  | Referent | | | | Referent | | | |
| <1mo |  |  |  |  |  |  |  |  | 1.45 | 0.25 | 2.66 | 0.018 | 0.57 | -0.61 | 1.74 | 0.340 |
| 1 to <3mo |  |  |  |  |  |  |  |  | 2.35 | 1.14 | 3.56 | <0.001 | 1.17 | -0.01 | 2.35 | 0.051 |
| 3 to <6mo |  |  |  |  |  |  |  |  | 3.32 | 2.06 | 4.58 | <0.001 | 1.41 | 0.17 | 2.64 | 0.025 |
| ≥6 |  |  |  |  |  |  |  |  | 4.34 | 2.98 | 5.70 | <0.001 | 2.29 | 0.96 | 3.62 | 0.001 |
| No alcohol in pregnancy |  |  |  |  |  |  |  |  | Referent | | | | Referent | | | |
| 1-7 drinks/wk |  |  |  |  |  |  |  |  | 1.05 | -0.11 | 2.21 | 0.074 | 0.07 | -1.08 | 1.21 | 0.910 |
| >7 drinks/wk |  |  |  |  |  |  |  |  | 1.4 | -0.36 | 3.10 | 0.120 | 0.51 | -1.25 | 2.26 | 0.568 |
| Maternal pre-pregnancy BMI |  |  |  |  |  |  |  |  | -0.27 | -0.36 | -0.17 | <0.001 | -0.24 | -0.33 | -0.14 | <0.001 |
| Partnered |  |  |  |  |  |  |  |  |  |  |  |  | 0.67 | -0.31 | 1.64 | 0.182 |
| Social Class (I & II) |  |  |  |  |  |  |  |  |  |  |  |  | Referent | | | |
| III |  |  |  |  |  |  |  |  |  |  |  |  | -1.06 | -1.97 | -0.15 | 0.023 |
| IV & V |  |  |  |  |  |  |  |  |  |  |  |  | -2.39 | -3.86 | -0.92 | 0.002 |
| Maternal Education (CSE + vocational) |  |  |  |  |  |  |  |  |  |  |  |  | Referent | | | |
| O level |  |  |  |  |  |  |  |  |  |  |  |  | 1.98 | 0.97 | 3.00 | <0.001 |
| A level |  |  |  |  |  |  |  |  |  |  |  |  | 3.15 | 1.86 | 4.43 | <0.001 |
| ≥Degree |  |  |  |  |  |  |  |  |  |  |  |  | 7.32 | 5.71 | 8.93 | <0.001 |
| Other children (none) |  |  |  |  |  |  |  |  |  |  |  |  | Referent | | | |
| 1 |  |  |  |  |  |  |  |  |  |  |  |  | -0.66 | -1.79 | 0.47 | 0.254 |
| ≥2 |  |  |  |  |  |  |  |  |  |  |  |  | -1.93 | -4.03 | 0.17 | 0.072 |
| Family Income (<£200) |  |  |  |  |  |  |  |  |  |  |  |  | Referent | | | |
| £ 200-399 |  |  |  |  |  |  |  |  |  |  |  |  | 1.13 | 0.12 | 2.15 | 0.029 |
| £ >400 |  |  |  |  |  |  |  |  |  |  |  |  | 2.97 | 1.91 | 4.03 | <0.001 |
| Maternal smoking (never) |  |  |  |  |  |  |  |  |  |  |  |  | Referent | | | |
| Quit |  |  |  |  |  |  |  |  |  |  |  |  | 0.13 | -0.64 | 0.90 | 0.742 |
| In pregnancy |  |  |  |  |  |  |  |  |  |  |  |  | 0.17 | -0.96 | 1.30 | 0.768 |
| HOME |  |  |  |  |  |  |  |  |  |  |  |  | 0.16 | -0.01 | 0.33 | 0.058 |
| Maternal IQ |  |  |  |  |  |  |  |  |  |  |  |  | 0.19 | 0.15 | 0.28 | <0.001 |

Abbreviations: CI, confidence interval; CSE Certificate of Secondary Education; IQ intelligence quotient

^1^ Eligible participants were children who had at least one measurement of IQ collected at either 8 or 15 years of age (n=7652). IQ was measured using the Wechsler Intelligence Scale for Children at 8 years of age. Incomplete IQ or missing covariable data was imputed by Multiple Imputation.

^2^ Analyses show the beta-coefficient from multivariable linear regression analysis using generalized linear models. Model 1 is the association between full scale IQ at 8 years of age (outcome) and dietary pattern trajectory intercept and slope (predictors). Model 2: model 1 plus adjustment for all other dietary pattern trajectories and intercepts. Model 3: model 2 plus adjustment for the following perinatal variables; sex, gestational age at birth, birth weight, ethnicity, singleton/twin, maternal age and parity. Model 4: model 3 plus adjustment for the following sociodemographic variables; social class (according to standard UK classifications of occupation at the time of birth[3]), maternal education, other children, family income, maternal smoking, stimulation in the home environment (using an adaptation of the HOME questionnaire[4]) and maternal IQ (measured by WASI when the study child was 15 years of age).

^3^ Beta coefficients are scaled to reflect the change in dietary patterns over 6 to 24 months of age.

**Table S3:** Associations between dietary pattern trajectories measured from 6 to 24 months and verbal IQ^1^ at 8 years of age (n=7652)^1^

|  | Model 1^2^ | | | | | Model 2 | | | | Model 3 | | | | Model 4 | | | |
| --- | --- | --- | --- | --- | --- | --- | --- | --- | --- | --- | --- | --- | --- | --- | --- | --- | --- |
|  | β^3^ | 95% CI | | | p | β^3^ | 95% CI | | p | β^3^ | 95% CI | | p | β^3^ | 95% CI | | p |
|  |  | Lower | | Upper |  |  | Lower | Upper |  |  | Lower | Upper |  |  | Lower | Upper |  |
| Healthy Intercept | 6.39 | 5.69 | 7.08 | | <0.001 | 5.06 | 4.34 | 5.78 | <0.001 | 2.70 | 1.82 | 3.59 | <0.001 | 1.00 | 0.08 | 1.92 | 0.033 |
| Healthy slope | 5.20 | 4.33 | 6.06 | | 0.006 | 3.78 | 2.86 | 4.70 | <0.001 | 2.66 | 1.72 | 3.61 | <0.001 | 0.95 | 0.02 | 1.88 | 0.046 |
| Discretionary Intercept | -7.03 | -7.96 | -6.09 | | <0.001 | -5.68 | -6.67 | -4.69 | <0.001 | -3.39 | -4.45 | -2.32 | <0.001 | -1.51 | -2.56 | -0.45 | 0.006 |
| Discretionary slope | -3.03 | -3.65 | -2.41 | | <0.001 | -2.01 | -2.70 | -1.33 | <0.001 | -1.30 | -1.96 | -0.65 | <0.001 | -0.52 | -1.16 | 0.12 | 0.110 |
| Traditional Intercept | 2.89 | 1.56 | 4.22 | | <0.001 | 2.22 | 0.86 | 3.58 | 0.002 | 1.63 | 0.27 | 2.99 | 0.020 | 0.97 | -0.46 | 2.19 | 0.196 |
| Traditional slope | -0.89 | -1.50 | -0.28 | | 0.005 | -0.20 | -0.78 | 0.39 | 0.502 | -0.12 | -0.68 | 0.45 | 0.682 | -0.11 | -0.64 | 0.41 | 0.666 |
| Ready-to-eat Intercept | 17.84 | -1.66 | 37.34 | | 0.071 | 11.04 | -1.44 | 23.53 | 0.081 | 5.32 | -4.54 | 15.19 | 0.280 | -2.61 | -9.38 | 4.15 | 0.444 |
| Ready-to-eat slope | 22.74 | 3.32 | 42.15 | | 0.024 | 13.02 | 1.00 | 25.04 | 0.035 | 9.01 | -0.58 | 18.61 | 0.064 | 2.42 | -3.51 | 8.35 | 0.414 |
| Female |  |  |  | |  |  |  |  |  | -1.03 | -1.74 | -0.32 | 0.005 | 1.00 | 0.08 | 1.92 | 0.001 |
| Gestational age at birth (wk) 37-42 |  |  |  | |  |  |  |  |  | Referent | | | | Referent | | | |
| <33 |  |  |  | |  |  |  |  |  | -0.90 | -4.67 | 2.87 | 0.638 | -0.81 | -4.45 | 2.83 | 0.661 |
| 33-36 |  |  |  | |  |  |  |  |  | 1.33 | -0.58 | 3.14 | 0.149 | 1.33 | -0.45 | 3.11 | 0.142 |
| >42 |  |  |  | |  |  |  |  |  | 2.70 | -2.53 | 7.93 | 0.311 | 3.21 | -1.89 | 8.30 | 0.217 |
| Birth weight (kg) |  |  |  | |  |  |  |  |  | 2.37 | 1.58 | 3.15 | <0.001 | 1.87 | 1.11 | 2.64 | <0.001 |
| White Ethnicity |  |  |  | |  |  |  |  |  | 0.97 | -0.80 | 2.74 | 0.281 | 0.16 | -1.57 | 1.88 | 0.858 |
| Twin |  |  |  | |  |  |  |  |  | -1.40 | -3.70 | 0.89 | 0.231 | -1.20 | -3.60 | 1.21 | 0.329 |
| Maternal age (y) |  |  |  | |  |  |  |  |  | 0.54 | 0.45 | 0.63 | <0.001 | 0.29 | 0.20 | 0.38 | <0.001 |
| Parity |  |  |  | |  |  |  |  |  | -2.80 | -3.25 | -2.34 | <0.001 | -1.26 | -2.17 | -0.35 | 0.007 |
| Breastfed (never) |  |  |  | |  |  |  |  |  | Referent | | | | Referent | | | |
| <1mo |  |  |  | |  |  |  |  |  | 1.18 | -0.03 | 2.39 | 0.056 | 0.33 | -0.85 | 1.51 | 0.586 |
| 1 to <3mo |  |  |  | |  |  |  |  |  | 1.59 | 0.34 | 2.84 | 0.013 | 0.46 | -0.75 | 1.67 | 0.456 |
| 3 to <6mo |  |  |  | |  |  |  |  |  | 3.01 | 1.71 | 4.31 | <0.001 | 1.19 | -0.08 | 2.47 | 0.066 |
| ≥6 |  |  |  | |  |  |  |  |  | 4.01 | 2.58 | 5.44 | <0.001 | 2.10 | 0.70 | 3.50 | 0.003 |
| No alcohol in pregnancy |  |  |  | |  |  |  |  |  | Referent | | | | Referent | | | |
| 1-7 drinks/wk |  |  |  | |  |  |  |  |  | 1.23 | 0.03 | 2.44 | 0.045 | 0.26 | -0.92 | 1.45 | 0.659 |
| >7 drinks/wk |  |  |  | |  |  |  |  |  | 1.52 | -0.24 | 3.28 | 0.090 | 0.64 | -1.10 | 2.38 | 0.467 |
| Maternal pre-preg BMI |  |  |  | |  |  |  |  |  | -0.18 | -0.28 | -0.09 | <0.001 | -0.15 | -0.25 | -0.05 | 0.003 |
| Partnered |  |  |  | |  |  |  |  |  |  |  |  |  | 0.75 | -0.23 | 1.73 | 0.132 |
| Social Class (I & II) |  |  |  | |  |  |  |  |  |  |  |  |  | Referent | | | |
| III |  |  |  | |  |  |  |  |  |  |  |  |  | -1.23 | -2.12 | -0.35 | 0.006 |
| IV & V |  |  |  | |  |  |  |  |  |  |  |  |  | -2.63 | -4.10 | -1.20 | <0.001 |
| Maternal Education (CSE + vocational) |  |  |  | |  |  |  |  |  |  |  |  |  | Referent | | | |
| O level |  |  |  | |  |  |  |  |  |  |  |  |  | 2.27 | 1.21 | 3.32 | <0.001 |
| A level |  |  |  | |  |  |  |  |  |  |  |  |  | 3.48 | 2.16 | 4.79 | <0.001 |
| ≥Degree |  |  |  | |  |  |  |  |  |  |  |  |  | 7.40 | 5.75 | 9.06 | <0.001 |
| Other children (none) |  |  |  | |  |  |  |  |  |  |  |  |  | Referent | | | |
| 1 |  |  |  | |  |  |  |  |  |  |  |  |  | -1.01 | -2.20 | 0.17 | 0.093 |
| ≥2 |  |  |  | |  |  |  |  |  |  |  |  |  | -2.28 | -4.47 | -0.10 | 0.041 |
| Family Income (<£200) |  |  |  | |  |  |  |  |  |  |  |  |  | Referent | | | |
| £ 200-399 |  |  |  | |  |  |  |  |  |  |  |  |  | 1.03 | -0.04 | 2.11 | 0.060 |
| £ >400 |  |  |  | |  |  |  |  |  |  |  |  |  | 3.02 | 1.88 | 4.15 | <0.001 |
| Maternal smoking (never) |  |  |  | |  |  |  |  |  |  |  |  |  | Referent | | | |
| Quit |  |  |  | |  |  |  |  |  |  |  |  |  | 0.20 | -0.60 | 0.99 | 0.629 |
| In pregnancy |  |  |  | |  |  |  |  |  |  |  |  |  | 0.62 | -0.51 | 1.74 | 0.280 |
| HOME |  |  |  | |  |  |  |  |  |  |  |  |  | 0.12 | -0.06 | 0.29 | 0.196 |
| Maternal IQ |  |  |  | |  |  |  |  |  |  |  |  |  | 0.15 | 0.11 | 0.20 | <0.001 |

Abbreviations: CI, confidence interval; CSE Certificate of Secondary Education; IQ intelligence quotient

^1^ Eligible participants were children who had IQ measured at either 8 or 15 years of age (n=7652). IQ was measured using the Wechsler Intelligence Scale for Children at 8 years. Incomplete IQ or missing covariable data was imputed by Multiple Imputation.

^2^ Analyses show the beta-coefficient from multivariable linear regression analysis using generalized linear models. Model 1 is the association between full scale IQ at 8 years of age (outcome) and dietary pattern trajectory intercept and slope (predictors). Model 2: model 1 plus adjustment for all other dietary pattern trajectories and intercepts. Model 3: model 2 plus adjustment for the following perinatal variables; sex, gestational age at birth, birth weight, ethnicity, singleton/twin, maternal age and parity. Model 4: model 3 plus adjustment for the following sociodemographic variables; social class (according to standard UK classifications of occupation at the time of birth[3]), maternal education, other children, family income, maternal smoking, stimulation in the home environment (using an adaptation of the HOME questionnaire[4]) and maternal IQ (measured by WASI when the study child was 15 years of age).

^3^ Beta coefficients are scaled to reflect the change in dietary patterns over 6 to 24 months of age.

**Table S4:** Associations between dietary pattern trajectories measured from 6 to 24 months and performance IQ^1^ at 8 years of age (n=7652)^1^

|  | Model 1^2^ | | | | Model 2 | | | | Model 3 | | | | Model 4 | | | |
| --- | --- | --- | --- | --- | --- | --- | --- | --- | --- | --- | --- | --- | --- | --- | --- | --- |
|  | β^3^ | 95% CI | | p | β^3^ | 95% CI | | p | β^3^ | 95% CI | | p | β^3^ | 95% CI | | p |
|  |  | Lower | Upper |  |  | Lower | Upper |  |  | Lower | Upper |  |  | Lower | Upper |  |
| Healthy Intercept | 4.21 | 3.53 | 4.88 | <0.001 | 3.37 | 5.67 | 4.07 | <0.001 | 1.57 | 0.67 | 2.47 | 0.001 | 0.03 | -0.86 | 0.93 | 0.939 |
| Healthy slope | 4.02 | 3.06 | 4.99 | <0.001 | 3.11 | 2.13 | 4.10 | <0.001 | 2.49 | 1.46 | 3.52 | <0.001 | 1.03 | -0.01 | 2.06 | 0.051 |
| Discretionary Intercept | -3.93 | -4.97 | -2.89 | <0.001 | -3.19 | -4.29 | -2.09 | <0.001 | -1.60 | -2.82 | -0.39 | 0.010 | 0.03 | -1.20 | 1.26 | 0.959 |
| Discretionary slope | -1.89 | -2.63 | -1.14 | <0.001 | -1.23 | -2.07 | -0.39 | 0.005 | -0.79 | -1.64 | 0.05 | 0.066 | -0.10 | -0.94 | 0.73 | 0.802 |
| Traditional Intercept | 2.72 | 1.54 | 3.91 | <0.001 | 2.13 | 0.91 | 3.36 | 0.001 | 1.72 | 0.47 | 2.96 | 0.007 | 0.98 | -0.26 | 2.23 | 0.121 |
| Traditional slope | -0.61 | -1.19 | -0.02 | 0.043 | -0.22 | -0.83 | 0.39 | 0.466 | -0.19 | -0.77 | 0.40 | 0.527 | -0.20 | -0.79 | 0.39 | 0.495 |
| Ready-to-eat Intercept | 10.36 | -1.92 | 22.64 | 0.095 | 5.56 | -2.96 | 14.08 | 0.196 | 2.53 | -5.50 | 10.56 | 0.531 | -4.42 | -12.21 | 3.38 | 0.262 |
| Ready-to-eat slope | 12.30 | 1.68 | 22.92 | 0.025 | 5.85 | -0.73 | 12.42 | 0.080 | 3.33 | -2.65 | 9.31 | 0.268 | -2.52 | -8.53 | 3.50 | 0.404 |
| Female |  |  |  |  |  |  |  |  | 1.35 | 0.59 | 2.11 | <0.001 | 1.25 | 0.51 | 1.99 | 0.001 |
| Gestational age at birth (wk); 37-42 |  |  |  |  |  |  |  |  | Referent | | | | Referent | | | |
| <33 |  |  |  |  |  |  |  |  | -3.18 | -7.20 | 0.83 | 0.120 | -3.23 | -7.21 | 0.74 | 0.111 |
| 33-36 |  |  |  |  |  |  |  |  | 2.19 | 0.26 | 4.12 | 0.026 | 2.14 | 0.22 | 4.06 | 0.029 |
| >42 |  |  |  |  |  |  |  |  | -2.22 | -7.84 | 3.41 | 0.440 | -1.81 | -7.35 | 3.72 | 0.521 |
| Birth weight (kg) |  |  |  |  |  |  |  |  | 2.69 | 1.87 | 3.51 | <0.001 | 2.15 | 1.34 | 2.97 | <0.001 |
| White Ethnicity |  |  |  |  |  |  |  |  | 2.31 | 042 | 4.20 | 0.017 | 1.52 | -0.37 | 3.41 | 0.115 |
| Twin |  |  |  |  |  |  |  |  | -3.10 | -5.53 | -0.67 | 0.012 | -3.26 | -5.81 | -0.70 | 0.012 |
| Maternal age (y) |  |  |  |  |  |  |  |  | 0.26 | 0.16 | 0.36 | <0.001 | 0.04 | -0.06 | 0.14 | 0.401 |
| Parity |  |  |  |  |  |  |  |  | -1.68 | -2.16 | -1.19 | <0.001 | -0.63 | -1.62 | 0.36 | 0.212 |
| Breastfed (never) |  |  |  |  |  |  |  |  | Referent | | | | Referent | | | |
| <1mo |  |  |  |  |  |  |  |  | 1.41 | 0.08 | 2.74 | 0.038 | 0.69 | -0.62 | 2.01 | 0.301 |
| 1 to <3mo |  |  |  |  |  |  |  |  | 2.61 | 1.29 | 3.93 | <0.001 | 1.66 | 0.34 | 2.97 | 0.013 |
| 3 to <6mo |  |  |  |  |  |  |  |  | 2.90 | 1.52 | 4.29 | <0.001 | 1.34 | -0.05 | 2.73 | 0.058 |
| ≥6 |  |  |  |  |  |  |  |  | 3.74 | 2.22 | 5.26 | <0.001 | 2.03 | 0.51 | 3.56 | 0.009 |
| No alcohol in pregnancy |  |  |  |  |  |  |  |  | Referent | | | | Referent | | | |
| 1-7 drinks/wk |  |  |  |  |  |  |  |  | 0.54 | -0.62 | 1.71 | 0.352 | -0.21 | -1.38 | 0.96 | 0.720 |
| >7 drinks/wk |  |  |  |  |  |  |  |  | 0.82 | -1.05 | 2.69 | 0.388 | 0.19 | -1.73 | 2.12 | 0.842 |
| Maternal pre-preg BMI |  |  |  |  |  |  |  |  | -0.29 | -0.40 | -0.19 | <0.001 | -0.27 | -0.37 | -0.16 | <0.001 |
| Partnered |  |  |  |  |  |  |  |  |  |  |  |  | 0.46 | -0.65 | 1.56 | 0.419 |
| Social Class (I & II) |  |  |  |  |  |  |  |  |  |  |  |  | Referent | | | |
| III |  |  |  |  |  |  |  |  |  |  |  |  | -0.53 | -1.59 | 0.52 | 0.320 |
| IV & V |  |  |  |  |  |  |  |  |  |  |  |  | -1.45 | -3.11 | 0.22 | 0.088 |
| Maternal Education (CSE + vocational) |  |  |  |  |  |  |  |  |  |  |  |  | Referent | | | |
| O level |  |  |  |  |  |  |  |  |  |  |  |  | 1.30 | 0.17 | 2.43 | 0.025 |
| A level |  |  |  |  |  |  |  |  |  |  |  |  | 2.09 | 0.71 | 3.48 | 0.003 |
| ≥Degree |  |  |  |  |  |  |  |  |  |  |  |  | 5.62 | 3.85 | 7.40 | <0.001 |
| Other children (none) |  |  |  |  |  |  |  |  |  |  |  |  | Referent | | | |
| 1 |  |  |  |  |  |  |  |  |  |  |  |  | -0.19 | -1.48 | 1.11 | 0.779 |
| ≥2 |  |  |  |  |  |  |  |  |  |  |  |  | -1.30 | -3.70 | 1.10 | 0.289 |
| Family Income (<£200) |  |  |  |  |  |  |  |  |  |  |  |  | Referent | | | |
| £ 200-399 |  |  |  |  |  |  |  |  |  |  |  |  | 0.91 | -0.22 | 2.04 | 0.115 |
| £ >400 |  |  |  |  |  |  |  |  |  |  |  |  | 2.07 | 0.90 | 3.24 | 0.001 |
| Maternal smoking (never) |  |  |  |  |  |  |  |  |  |  |  |  | Referent | | | |
| Quit |  |  |  |  |  |  |  |  |  |  |  |  | 0.09 | -0.76 | 0.94 | 0.829 |
| In pregnancy |  |  |  |  |  |  |  |  |  |  |  |  | -0.38 | -1.64 | 0.88 | 0.552 |
| HOME |  |  |  |  |  |  |  |  |  |  |  |  | 0.19 | 0.003 | 0.37 | 0.047 |
| Maternal IQ |  |  |  |  |  |  |  |  |  |  |  |  | 0.18 | 0.13 | 0.22 | <0.001 |

Abbreviations: CI, confidence interval; CSE Certificate of Secondary Education; IQ intelligence quotient

^1^ Eligible participants were children who had at least one measurement of IQ collected at either 8 or 15 years of age (n=7652). IQ was measured using the Wechsler Intelligence Scale for Children. Incomplete IQ or missing covariable data was imputed by Multiple Imputation.

^2^ Analyses show the beta-coefficient from multivariable linear regression analysis using generalized linear models. Model 1 is the association between full scale IQ at 8 years of age (outcome) and dietary pattern trajectory intercept and slope (predictors). Model 2: model 1 plus adjustment for all other dietary pattern trajectories and intercepts. Model 3: model 2 plus adjustment for the following perinatal variables; sex, gestational age at birth, birth weight, ethnicity, singleton/twin, maternal age and parity. Model 4: model 3 plus adjustment for the following sociodemographic variables; social class (according to standard UK classifications of occupation at the time of birth[3]), maternal education, other children, family income, maternal smoking, stimulation in the home environment (using an adaptation of the HOME questionnaire[4]) and maternal IQ (measured by WASI when the study child was 15 years of age).

^3^ Beta coefficients are scaled to reflect the change in dietary patterns over 6 to 24 months of age.

**Table S5:** Associations between dietary pattern trajectories measured from 6 to 24 months and full scale IQ^1^ at 15 years of age (n=7652)^1^

|  | Model 1^2^ | | | | Model 2 | | | | Model 3 | | | | Model 4 | | | |
| --- | --- | --- | --- | --- | --- | --- | --- | --- | --- | --- | --- | --- | --- | --- | --- | --- |
|  | β^3^ | 95% CI | | p | β^3^ | 95% CI | | p | β^3^ | 95% CI | | p | β^3^ | 95% CI | | p |
|  |  | Lower | Upper |  |  | Lower | Upper |  |  | Lower | Upper |  |  | Lower | Upper |  |
| Healthy Intercept | 4.44 | 3.92 | 4.96 | <0.001 | 3.51 | 2.94 | 4.09 | <0.001 | 2.02 | 1.28 | 2.76 | <0.001 | 0.75 | 0.00 | 1.50 | 0.049 |
| Healthy slope | 3.08 | 2.37 | 3.79 | <0.001 | 2.41 | 1.66 | 3.15 | <0.001 | 1.71 | 0.92 | 2.49 | <0.001 | 0.49 | -0.28 | 1.26 | 0.208 |
| Discretionary Intercept | -4.00 | -4.80 | -3.21 | <0.001 | -3.17 | -4.00 | -2.35 | <0.001 | -1.68 | -2.52 | -0.84 | <0.001 | -0.29 | -1.14 | 0.56 | 0.503 |
| Discretionary slope | -2.57 | -3.11 | -2.03 | <0.001 | -1.81 | -2.40 | -1.22 | <0.001 | -1.34 | -1.96 | -0.72 | <0.001 | -0.73 | -1.33 | -0.14 | 0.017 |
| Traditional Intercept | 1.30 | 0.33 | 2.28 | 0.010 | 0.85 | -0.15 | 1.85 | 0.047 | 0.56 | -0.42 | 1.54 | 0.257 | 0.09 | -0.92 | 1.09 | 0.860 |
| Traditional slope | -0.98 | -1.40 | -0.56 | <0.001 | -0.44 | -0.84 | -0.05 | 0.041 | -0.39 | -0.79 | 0.00 | 0.049 | -0.41 | -0.77 | -0.04 | 0.031 |
| Ready-to-eat Intercept | 12.48 | -0.39 | 25.35 | 0.057 | 9.27 | 0.14 | 18.41 | 0.095 | 5.56 | -2.14 | 13.26 | 0.151 | -0.14 | -5.87 | 5.60 | 0.962 |
| Ready-to-eat slope | 14.14 | 1.76 | 26.52 | 0.027 | 8.58 | 0.37 | 16.80 | 0.029 | 5.90 | -0.89 | 12.69 | 0.086 | 1.11 | -3.10 | 5.33 | 0.597 |
| Female |  |  |  |  |  |  |  |  | -0.87 | -1.44 | -0.30 | 0.003 | -0.95 | -1.52 | -0.39 | 0.001 |
| Gestational age at birth (wk); 37-42 |  |  |  |  |  |  |  |  | Referent | | | | Referent | | | |
| <33 |  |  |  |  |  |  |  |  | -0.20 | -3.43 | 3.02 | 0.900 | -0.19 | -3.38 | 3.00 | 0.906 |
| 33-36 |  |  |  |  |  |  |  |  | 1.93 | 0.49 | 3.37 | 0.009 | 1.92 | 0.48 | 3.35 | 0.009 |
| >42 |  |  |  |  |  |  |  |  | -1.23 | -6.00 | 3.54 | 0.609 | -1.13 | -5.68 | 3.41 | 0.623 |
| Birth weight (kg) |  |  |  |  |  |  |  |  | 1.00 | 0.34 | 1.66 | 0.003 | 0.61 | -0.04 | 1.27 | 0.067 |
| White Ethnicity |  |  |  |  |  |  |  |  | -0.06 | -1.48 | 1.36 | 0.935 | -0.65 | -2.09 | 0.78 | 0.371 |
| Twin |  |  |  |  |  |  |  |  | -1.10 | -3.01 | 0.80 | 0.255 | -1.23 | -3.23 | 0.77 | 0.228 |
| Maternal age (y) |  |  |  |  |  |  |  |  | 0.33 | 0.26 | 0.41 | <0.001 | 0.15 | 0.07 | 0.22 | <0.001 |
| Parity |  |  |  |  |  |  |  |  | -1.41 | -1.79 | -1.02 | <0.001 | -0.51 | -1.26 | 0.24 | 0.180 |
| Breastfed (never) |  |  |  |  |  |  |  |  | Referent | | | | Referent | | | |
| <1mo |  |  |  |  |  |  |  |  | 0.98 | 0.02 | 1.94 | 0.047 | 0.39 | -0.55 | 1.34 | 0.414 |
| 1 to <3mo |  |  |  |  |  |  |  |  | 1.72 | 0.76 | 2.68 | <0.001 | 0.95 | 0.03 | 1.88 | 0.043 |
| 3 to <6mo |  |  |  |  |  |  |  |  | 2.41 | 1.41 | 3.42 | <0.001 | 1.09 | 0.11 | 2.08 | 0.029 |
| ≥6 |  |  |  |  |  |  |  |  | 2.80 | 1.68 | 3.92 | <0.001 | 1.37 | 0.22 | 2.51 | 0.019 |
| No alcohol in pregnancy |  |  |  |  |  |  |  |  | Referent | | | | Referent | | | |
| 1-7 drinks/wk |  |  |  |  |  |  |  |  | 0.87 | -0.02 | 1.77 | 0.056 | 0.16 | -0.72 | 1.04 | 0.719 |
| >7 drinks/wk |  |  |  |  |  |  |  |  | 0.97 | -0.40 | 2.34 | 0.163 | 0.31 | -1.07 | 1.69 | 0.660 |
| Maternal pre-preg BMI |  |  |  |  |  |  |  |  | -0.18 | -0.27 | -0.09 | <0.001 | -0.16 | -0.25 | -0.08 | <0.001 |
| Partnered |  |  |  |  |  |  |  |  |  |  |  |  | 0.27 | -0.60 | 1.14 | 0.537 |
| Social Class (I & II) |  |  |  |  |  |  |  |  |  |  |  |  | Referent | | | |
| III |  |  |  |  |  |  |  |  |  |  |  |  | -0.58 | -1.32 | 0.16 | 0.123 |
| IV & V |  |  |  |  |  |  |  |  |  |  |  |  | -0.33 | -1.60 | 0.93 | 0.600 |
| Maternal Education (CSE + vocational) |  |  |  |  |  |  |  |  |  |  |  |  | Referent | | | |
| O level |  |  |  |  |  |  |  |  |  |  |  |  | 0.46 | -0.35 | 1.27 | 0.268 |
| A level |  |  |  |  |  |  |  |  |  |  |  |  | 1.60 | 0.59 | 2.61 | 0.002 |
| ≥Degree |  |  |  |  |  |  |  |  |  |  |  |  | 3.90 | 2.62 | 5.17 | <0.001 |
| Other children (none) |  |  |  |  |  |  |  |  |  |  |  |  | Referent | | | |
| 1 |  |  |  |  |  |  |  |  |  |  |  |  | -0.82 | -1.74 | 0.09 | 0.078 |
| ≥2 |  |  |  |  |  |  |  |  |  |  |  |  | -1.30 | -3.06 | 0.45 | 0.143 |
| Family Income (<£200) |  |  |  |  |  |  |  |  |  |  |  |  | Referent | | | |
| £ 200-399 |  |  |  |  |  |  |  |  |  |  |  |  | 0.58 | -0.34 | 1.49 | 0.212 |
| £ >400 |  |  |  |  |  |  |  |  |  |  |  |  | 2.06 | 1.13 | 3.00 | <0.001 |
| Maternal smoking (never) |  |  |  |  |  |  |  |  |  |  |  |  | Referent | | | |
| Quit |  |  |  |  |  |  |  |  |  |  |  |  | 0.26 | -0.41 | 0.93 | 0.451 |
| In pregnancy |  |  |  |  |  |  |  |  |  |  |  |  | 0.16 | -0.82 | 1.13 | 0.748 |
| HOME |  |  |  |  |  |  |  |  |  |  |  |  | -0.01 | -0.15 | 0.14 | 0.913 |
| Maternal IQ |  |  |  |  |  |  |  |  |  |  |  |  | 0.17 | 0.15 | 0.20 | <0.001 |

Abbreviations: CI, confidence interval; CSE Certificate of Secondary Education; IQ intelligence quotient

^1^ Eligible participants were children who had at least one measurement of IQ collected at either 8 or 15 years of age (n=7652). IQ was measured using the Wechsler Abbreviated Scale of Intelligence at 15 years of age. Incomplete IQ or missing covariable data was imputed by Multiple Imputation.

^2^ Analyses show the beta-coefficient from multivariable linear regression analysis using generalized linear models. Model 1 is the association between full scale IQ at 15 years of age (outcome) and dietary pattern trajectory intercept and slope (predictors). Model 2: model 1 plus adjustment for all other dietary pattern trajectories and intercepts. Model 3: model 2 plus adjustment for the following perinatal variables; sex, gestational age at birth, birth weight, ethnicity, singleton/twin, maternal age and parity. Model 4: model 3 plus adjustment for the following sociodemographic variables; social class (according to standard UK classifications of occupation at the time of birth[3]), maternal education, other children, family income, maternal smoking, stimulation in the home environment (using an adaptation of the HOME questionnaire[4]) and maternal IQ (measured by WASI when the study child was 15 years of age).

^3^ Beta coefficients are scaled to reflect the change in dietary patterns over 6 to 24 months of age.

**Table S6:** Complete case analysis of the associations between dietary pattern trajectories measured from 6 to 24 months and Full Scale IQ^1^ at 8 years of age (n=950)^1^

|  | Model 1^2^ | | | | Model 2 | | | | Model 3 | | | | Model 4 | | | |
| --- | --- | --- | --- | --- | --- | --- | --- | --- | --- | --- | --- | --- | --- | --- | --- | --- |
|  | β^3^ | 95% CI | | p | β^3^ | 95% CI | | p | β^3^ | 95% CI | | p | β^3^ | 95% CI | | p |
|  |  | Lower | Upper |  |  | Lower | Upper |  |  | Lower | Upper |  |  | Lower | Upper |  |
| Healthy Intercept | 5.25 | 3.57 | 6.93 | <0.001 | 4.12 | 2.40 | 5.83 | <0.001 | 2.57 | 0.54 | 4.61 | 0.013 | 0.85 | -1.18 | 2.88 | 0.411 |
| Healthy slope | 0.24 | 1.21 | 5.11 | 0.002 | 1.98 | 0.01 | 3.94 | 0.049 | 1.28 | -0.70 | 3.27 | 0.204 | 0.41 | -1.53 | 2.23 | 0.680 |
| Discretionary Intercept | -8.34 | -12.80 | -3.88 | <0.001 | -7.20 | -11.66 | -2.73 | 0.002 | -4.74 | -9.29 | -0.90 | 0.041 | -1.51 | -5.99 | 2.97 | 0.508 |
| Discretionary slope | -2.79 | -4.98 | 0.60 | 0.012 | -1.74 | -3.98 | 0.50 | 0.127 | -0.73 | -0.02 | 1.50 | 0.520 | 0.10 | -2.08 | 2.29 | 0.928 |
| Traditional Intercept | 3.02 | 0.01 | 6.03 | 0.049 | 2.81 | -0.19 | 5.81 | 0.067 | 2.32 | -69.31 | 5.30 | 0.127 | 1.40 | -1.53 | 4.33 | 0.348 |
| Traditional slope | -1.38 | -2.99 | 0.24 | 0.095 | -0.15 | -1.80 | 1.49 | 0.856 | -0.14 | -0.29 | 1.49 | 0.866 | -0.18 | -1.77 | 1.40 | 0.820 |
| Ready-to-eat Intercept | -1.41 | -11.75 | 8.94 | 0.079 | 0.76 | -9.48 | 10.99 | 0.885 | -0.94 | -118.69 | 9.38 | 0.858 | -6.23 | -16.30 | 3.82 | 0.224 |
| Ready-to-eat slope | 26.82 | 0.78 | 52.86 | 0.044 | 17.12 | -8.71 | 42.95 | 0.194 | 10.39 | -15.87 | 36.65 | 0.438 | -3.89 | -29.51 | 21.73 | 0.766 |
| Female |  |  |  |  |  |  |  |  | -0.35 | -2.34 | -1.64 | 0.729 | -0.66 | -2.59 | 1.26 | 0.499 |
| Gestational age at birth (wk); 37-42 |  |  |  |  |  |  |  |  | Referent | | | |  | | | |
| <33 |  |  |  |  |  |  |  |  | -10.72 | -26.34 | 4.90 | 0.178 | -11.33 | -26.48 | 3.82 | 0.143 |
| 33-36 |  |  |  |  |  |  |  |  | 1.62 | -3.46 | 6.69 | 0.532 | 0.61 | -4.32 | 5.54 | 0.809 |
| >42 |  |  |  |  |  |  |  |  | 4.16 | -17.38 | 25.70 | 0.705 | 5.97 | -14.90 | 26.83 | 0.575 |
| Birth weight (kg) |  |  |  |  |  |  |  |  | 2.37 | 0.15 | 4.59 | 0.037 | 1.59 | -0.59 | 3.78 | 0.152 |
| White Ethnicity |  |  |  |  |  |  |  |  | 1.95 | -4.51 | 8.41 | 0.553 | 1.37 | -4.93 | 7.67 | 0.670 |
| Twin |  |  |  |  |  |  |  |  | -4.26 | -10.77 | 2.25 | 0.199 | -3.62 | -10.84 | 3.59 | 0.324 |
| Maternal age (y) |  |  |  |  |  |  |  |  | 0.55 | 0.29 | 0.82 | <0.001 | 0.29 | 0.02 | 0.56 | 0.038 |
| Parity |  |  |  |  |  |  |  |  | -2.99 | -4.36 | -1.63 | <0.001 | -1.39 | -4.70 | 1.92 | 0.411 |
| Breastfed (never) |  |  |  |  |  |  |  |  | Referent | | | | Referent | | | |
| <1mo |  |  |  |  |  |  |  |  | 0.83 | -2.90 | 4.55 | 0.663 | -0.73 | -4.35 | 2.90 | 0.694 |
| 1 to <3mo |  |  |  |  |  |  |  |  | 0.72 | -3.02 | 4.47 | 0.705 | -0.58 | -4.22 | 3.06 | 0.755 |
| 3 to <6mo |  |  |  |  |  |  |  |  | 4.59 | 0.87 | 8.30 | 0.016 | 1.78 | -1.88 | 5.44 | 0.341 |
| ≥6 |  |  |  |  |  |  |  |  | 3.28 | -0.43 | 6.98 | 0.083 | 0.35 | -3.31 | 4.01 | 0.851 |
| No alcohol in pregnancy |  |  |  |  |  |  |  |  | Referent | | | | Referent | | | |
| 1-7 drinks/wk |  |  |  |  |  |  |  |  | 0.94 | -1.20 | 3.08 | 0.388 | -0.22 | -2.32 | 1.88 | 0.841 |
| >7 drinks/wk |  |  |  |  |  |  |  |  | 2.87 | -1.58 | 7.32 | 0.206 | 1.76 | -2.60 | 6.12 | 0.430 |
| Maternal pre-preg BMI |  |  |  |  |  |  |  |  | -0.24 | -0..52 | 0.04 | 0.091 | -0.25 | -0.52 | 0.03 | 0.075 |
| Partnered |  |  |  |  |  |  |  |  |  |  |  |  | 0.98 | -1.88 | 3.84 | 0.503 |
| Social Class (I & II) |  |  |  |  |  |  |  |  |  |  |  |  | Referent | | | |
| III |  |  |  |  |  |  |  |  |  |  |  |  | -0.67 | -2.97 | 1.63 | 0.568 |
| IV & V |  |  |  |  |  |  |  |  |  |  |  |  | 0.78 | -3.28 | 4.85 | 0.705 |
| Maternal Education (CSE + vocational) |  |  |  |  |  |  |  |  |  |  |  |  | Referent | | | |
| O level |  |  |  |  |  |  |  |  |  |  |  |  | 3.47 | 0.19 | 6.74 | 0.038 |
| A level |  |  |  |  |  |  |  |  |  |  |  |  | 3.49 | -0.15 | 7.13 | 0.060 |
| ≥Degree |  |  |  |  |  |  |  |  |  |  |  |  | 8.59 | 4.38 | 12.80 | <0.001 |
| Other children (none) |  |  |  |  |  |  |  |  |  |  |  |  | Referent | | | |
| 1 |  |  |  |  |  |  |  |  |  |  |  |  | -1.62 | -5.61 | 2.37 | 0.425 |
| ≥2 |  |  |  |  |  |  |  |  |  |  |  |  | -2.46 | -9.88 | 4.95 | 0.515 |
| Family Income (<£200) |  |  |  |  |  |  |  |  |  |  |  |  | Referent | | | |
| £ 200-399 |  |  |  |  |  |  |  |  |  |  |  |  | -1.65 | -5.61 | 2.37 | 0.425 |
| £ >400 |  |  |  |  |  |  |  |  |  |  |  |  | -2.46 | -9.88 | 4.95 | 0.515 |
| Maternal smoking (never) |  |  |  |  |  |  |  |  |  |  |  |  | Referent | | | |
| Quit |  |  |  |  |  |  |  |  |  |  |  |  | -0.69 | -2.94 | 1.56 | 0.549 |
| In pregnancy |  |  |  |  |  |  |  |  |  |  |  |  | -0.11 | -3.74 | 3.52 | 0.951 |
| HOME |  |  |  |  |  |  |  |  |  |  |  |  | 0.41 | -0.05 | 0.87 | 0.083 |
| Maternal IQ |  |  |  |  |  |  |  |  |  |  |  |  | 0.19 | 0.11 | 0.28 | <0.001 |

Abbreviations: CI, confidence interval; CSE Certificate of Secondary Education; IQ intelligence quotient

^1^ Eligible participants were children who had the Verbal and the Performance subscales of the Wechsler Intelligence Scale for Children measured at 8 years of age and had all dietary and covariable data.

^2^ Analyses show the beta-coefficient from multivariable linear regression analysis using generalized linear models. Model 1 is the association between full scale IQ at 8 years of age (outcome) and dietary pattern trajectory intercept and slope (predictors). Model 2: model 1 plus adjustment for all other dietary pattern trajectories and intercepts. Model 3: model 2 plus adjustment for the following perinatal variables; sex, gestational age at birth, birth weight, ethnicity, singleton/twin, maternal age and parity. Model 4: model 3 plus adjustment for the following sociodemographic variables; social class (according to standard UK classifications of occupation at the time of birth[3]), maternal education, other children, family income, maternal smoking, stimulation in the home environment (using an adaptation of the HOME questionnaire[4]) and maternal IQ (measured by WASI when the study child was 15 years of age).

^3^ Beta coefficients are scaled to reflect the change in dietary patterns over 6 to 24 months of age.

**Table S7:** Complete case analysis of the associations between dietary pattern trajectories measured from 6 to 24 months and verbal IQ^1^ at 8 years of age (n=954)^1^

|  | Model 1^2^ | | | | Model 2 | | | | Model 3 | | | | Model 4 | | | |
| --- | --- | --- | --- | --- | --- | --- | --- | --- | --- | --- | --- | --- | --- | --- | --- | --- |
|  | β^3^ | 95% CI | | p | β^3^ | 95% CI | | p | β^3^ | 95% CI | | p | β^3^ | 95% CI | | p |
|  |  | Lower | Upper |  |  | Lower | Upper |  |  | Lower | Upper |  |  | Lower | Upper |  |
| Healthy Intercept | 5.45 | 3.73 | 7.18 | <0.001 | 4.39 | 2.63 | 6.15 | <0.001 | 2.15 | 0.06 | 4.25 | 0.044 | 0.41 | -1.68 | 2.51 | 0.700 |
| Healthy slope | 2.88 | 1.00 | 4.77 | 0.003 | 1.88 | -0.03 | 3.78 | 0.053 | 1.48 | -0.44 | 3.40 | 0.130 | 0.72 | -1.15 | 2.60 | 0.450 |
| Discretionary Intercept | -8.58 | -13.19 | -3.97 | <0.001 | -7.45 | -12.08 | -2.83 | 0.002 | -4.54 | -9.24 | 0.16 | 0.058 | -1.44 | -6.09 | 3.21 | 0.542 |
| Discretionary slope | -2.56 | -4.80 | -0.33 | <0.001 | -1.50 | -3.79 | 0.78 | 0.197 | -0.53 | -2.81 | 1.75 | 0.647 | 0.24 | -2.00 | 2.48 | 0.831 |
| Traditional Intercept | 1.83 | -1.28 | 4.94 | 0.249 | 1.50 | -1.60 | 4.60 | 0.343 | 1.03 | -2.04 | 4.11 | 0.510 | 0.10 | -2.94 | 3.13 | 0.951 |
| Traditional slope | -1.41 | -3.02 | 0.21 | 0.088 | -0.29 | -1.93 | 1.35 | 0.729 | -0.22 | -1.85 | 1.41 | 0.794 | -0.26 | -1.85 | 1.33 | 0.747 |
| Ready-to-eat Intercept | 3.30 | -7.39 | 13.99 | 0.545 | 5.26 | -5.33 | 15.86 | 0.330 | 3.20 | -7.46 | 13.87 | 0.556 | -2.47 | -12.91 | 7.96 | 0.642 |
| Ready-to-eat slope | 37.74 | 10.93 | 64.54 | 0.006 | 28.54 | 1.92 | 55.16 | 0.036 | 21.78 | -5.26 | 48.82 | 0.114 | 7.00 | -19.51 | 33.51 | 0.604 |
| Female |  |  |  |  |  |  |  |  | -1.82 | -3.87 | 0.24 | 0.083 | -2.13 | -4.13 | -0.13 | 0.037 |
| Gestational age at birth (wk); 37-42 |  |  |  |  |  |  |  |  | Referent | | | | Referent | | | |
| <33 |  |  |  |  |  |  |  |  | -5.87 | -22.03 | 10.28 | 0.476 | -7.29 | -23.04 | 8.46 | 0.364 |
| 33-36 |  |  |  |  |  |  |  |  | -1.57 | -6.77 | 3.64 | 0.555 | -2.24 | -7.32 | 2.84 | 0.387 |
| >42 |  |  |  |  |  |  |  |  | 9.11 | -13.17 | 31.39 | 0.423 | 11.35 | -10.34 | 33.04 | 0.305 |
| Birth weight (kg) |  |  |  |  |  |  |  |  | 1.34 | -0.96 | 3.64 | 0.253 | 0.59 | -1.67 | 2.86 | 0.607 |
| White Ethnicity |  |  |  |  |  |  |  |  | 1.81 | -4.87 | 8.50 | 0.594 | 1.86 | -4.69 | 8.40 | 0.577 |
| Twin |  |  |  |  |  |  |  |  | -0.68 | -7.41 | 6.05 | 0.843 | 0.29 | -7.20 | 7.78 | 0.940 |
| Maternal age (y) |  |  |  |  |  |  |  |  | 0.57 | 0.30 | 0.85 | <0.001 | 0.32 | 0.04 | 0.60 | 0.025 |
| Parity |  |  |  |  |  |  |  |  | -3.1 | -4.51 | -1.69 | <0.001 | -1.06 | -4.50 | 2.38 | 0.545 |
| Breastfed (never) |  |  |  |  |  |  |  |  | Referent | | | | Referent | | | |
| <1mo |  |  |  |  |  |  |  |  | 1.06 | -2.76 | 4.89 | 0.585 | -0.33 | -4.07 | 3.41 | 0.862 |
| 1 to <3mo |  |  |  |  |  |  |  |  | -0.21 | -4.07 | 3.64 | 0.915 | -1.41 | -5.18 | 2.36 | 0.463 |
| 3 to <6mo |  |  |  |  |  |  |  |  | 5.03 | 1.20 | 8.86 | 0.010 | 2.22 | -1.57 | 6.01 | 0.250 |
| ≥6 |  |  |  |  |  |  |  |  | 5.15 | 1.33 | 8.97 | 0.008 | 2.42 | -1.36 | 6.22 | 0.209 |
| No alcohol in pregnancy |  |  |  |  |  |  |  |  | Referent | | | | Referent | | | |
| 1-7 drinks/wk |  |  |  |  |  |  |  |  | 1.62 | -0.58 | 3.82 | 0.150 | 0.40 | -1.78 | 2.57 | 0.719 |
| >7 drinks/wk |  |  |  |  |  |  |  |  | 0.83 | -3.78 | 5.43 | 0.725 | -0.82 | -5.25 | 3.82 | 0.757 |
| Maternal pre-preg BMI |  |  |  |  |  |  |  |  | -0.04 | -0.33 | 0.25 | 0.766 | -0.06 | -0.34 | 0.23 | 0.695 |
| Partnered |  |  |  |  |  |  |  |  |  |  |  |  | 0.83 | -2.14 | 3.80 | 0.584 |
| Social Class (I & II) |  |  |  |  |  |  |  |  |  |  |  |  | Referent | | | |
| III |  |  |  |  |  |  |  |  |  |  |  |  | -1.23 | -3.62 | 1.16 | 0.314 |
| IV & V |  |  |  |  |  |  |  |  |  |  |  |  | 0.25 | -3.97 | 4.48 | 0.906 |
| Maternal Education (CSE + vocational) |  |  |  |  |  |  |  |  |  |  |  |  | Referent | | | |
| O level |  |  |  |  |  |  |  |  |  |  |  |  | 4.11 | 0.72 | 7.50 | 0.017 |
| A level |  |  |  |  |  |  |  |  |  |  |  |  | 5.23 | 1.46 | 9.01 | 0.007 |
| ≥Degree |  |  |  |  |  |  |  |  |  |  |  |  | 9.17 | 4.80 | 13.54 | <0.001 |
| Other children (none) |  |  |  |  |  |  |  |  |  |  |  |  | Referent | | | |
| 1 |  |  |  |  |  |  |  |  |  |  |  |  | -1.73 | -5.87 | 2.41 | 0.413 |
| ≥2 |  |  |  |  |  |  |  |  |  |  |  |  | -3.65 | -11.35 | 4.06 | 0.353 |
| Family Income (<£200) |  |  |  |  |  |  |  |  |  |  |  |  | Referent | | | |
| £ 200-399 |  |  |  |  |  |  |  |  |  |  |  |  | -1.43 | -4.70 | 1.83 | 0.388 |
| £ >400 |  |  |  |  |  |  |  |  |  |  |  |  | 2.27 | -0.94 | 5.47 | 0.165 |
| Maternal smoking (never) |  |  |  |  |  |  |  |  |  |  |  |  | Referent | | | |
| Quit |  |  |  |  |  |  |  |  |  |  |  |  | -0.09 | -2.43 | 2.25 | 0.941 |
| In pregnancy |  |  |  |  |  |  |  |  |  |  |  |  | 2.11 | -1.66 | 5.88 | 0.272 |
| HOME |  |  |  |  |  |  |  |  |  |  |  |  | 0.34 | -0.14 | 0.83 | 0.159 |
| Maternal IQ |  |  |  |  |  |  |  |  |  |  |  |  | 0.17 | 0.08 | 0.27 | <0.001 |

Abbreviations: CI, confidence interval; CSE Certificate of Secondary Education; IQ intelligence quotient

^1^ Eligible participants were children who had the Verbal subscale of the Wechsler Intelligence Scale for Children measured at 8 years of age and had all dietary and covariable data.

^2^ Analyses show the beta-coefficient from multivariable linear regression analysis using generalized linear models. Model 1 is the association between full scale IQ at 8 years of age (outcome) and dietary pattern trajectory intercept and slope (predictors). Model 2: model 1 plus adjustment for all other dietary pattern trajectories and intercepts. Model 3: model 2 plus adjustment for the following perinatal variables; sex, gestational age at birth, birth weight, ethnicity, singleton/twin, maternal age and parity. Model 4: model 3 plus adjustment for the following sociodemographic variables; social class (according to standard UK classifications of occupation at the time of birth[3]), maternal education, other children, family income, maternal smoking, stimulation in the home environment (using an adaptation of the HOME questionnaire[4]) and maternal IQ (measured by WASI when the study child was 15 years of age).

^3^ Beta coefficients are scaled to reflect the change in dietary patterns over 6 to 24 months of age.

**Table S8:** Complete case analysis of the associations between dietary pattern trajectories measured from 6 to 24 months and performance IQ^1^ at 8 years of age (n=952)^1^

|  | Model 1^2^ | | | | Model 2 | | | | Model 3 | | | | Model 4 | | | |
| --- | --- | --- | --- | --- | --- | --- | --- | --- | --- | --- | --- | --- | --- | --- | --- | --- |
|  | β^3^ | 95% CI | | p | β^3^ | 95% CI | | p | β^3^ | 95% CI | | p | β^3^ | 95% CI | | p |
|  |  | Lower | Upper |  |  | Lower | Upper |  |  | Lower | Upper |  |  | Lower | Upper |  |
| Healthy Intercept | 3.90 | 2.11 | 5.68 | <0.001 | 3.02 | 1.19 | 4.86 | 0.001 | 2.31 | 0.13 | 4.50 | 0.038 | 1.00 | -1.24 | 3.19 | 0.387 |
| Healthy slope | 2.81 | 0.71 | 4.91 | 0.009 | 1.81 | -0.33 | 3.94 | 0.097 | 1.04 | -1.12 | 3.20 | 0.344 | 0.25 | -1.89 | 2.39 | 0.821 |
| Discretionary Intercept | -5.20 | -9.82 | -0.57 | <0.001 | -4.43 | -9.11 | 0.25 | 0.063 | -2.81 | -7.61 | 1.98 | 0.250 | 0.03 | -4.75 | 4.81 | 0.990 |
| Discretionary slope | -2.54 | -4.80 | -0.27 | <0.001 | -1.87 | -4.20 | 0.47 | 0.117 | -1.15 | -3.59 | 1.19 | 0.336 | -0.41 | -2.74 | 1.92 | 0.730 |
| Traditional Intercept | 4.59 | 1.42 | 7.75 | 0.005 | 4.58 | 1.37 | 7.79 | 0.005 | 4.26 | 1.06 | 7.47 | 0.009 | 3.56 | 0.36 | 6.75 | 0.029 |
| Traditional slope | -0.68 | -2.41 | 1.06 | 0.445 | 0.38 | -1.42 | 2.18 | 0.678 | 0.39 | -1.40 | 2.19 | 0.668 | 0.34 | -1.43 | 2.12 | 0.704 |
| Ready-to-eat Intercept | -5.62 | -16.55 | 5.31 | 0.313 | -3.94 | -14.88 | 7.00 | 0.480 | -4.60 | -15.68 | 6.48 | 0.415 | -8.97 | -19.93 | 1.98 | 0.108 |
| Ready-to-eat slope | 6.51 | -20.89 | 33.91 | 0.641 | -2.07 | -29.59 | 25.45 | 0.883 | -6.78 | -34.88 | 21.32 | 0.636 | -19.16 | -47.02 | 8.70 | 0.177 |
| Female |  |  |  |  |  |  |  |  | 1.77 | -0.37 | 3.91 | 0.105 | 1.46 | -0.64 | 3.47 | 0.172 |
| Gestational age at birth (wk); 37-42 |  |  |  |  |  |  |  |  | Referent | | | | Referent | | | |
| <33 |  |  |  |  |  |  |  |  | -13.15 | -29.94 | 3.64 | 0.124 | -12.81 | -29.36 | 3.73 | 0.129 |
| 33-36 |  |  |  |  |  |  |  |  | 4.30 | -1.15 | 9.76 | 0.122 | 3.36 | -2.02 | 8.75 | 0.221 |
| >42 |  |  |  |  |  |  |  |  | -2.15 | -25.31 | 21.01 | 0.856 | -0.82 | -23.60 | 21.96 | 0.944 |
| Birth weight (kg) |  |  |  |  |  |  |  |  | 3.18 | 0.79 | 5.56 | 0.009 | 2.51 | 0.13 | 4.89 | 0.039 |
| White Ethnicity |  |  |  |  |  |  |  |  | 1.35 | -5.59 | 8.30 | 0.702 | 0.24 | -6.63 | 7.12 | 0.945 |
| Twin |  |  |  |  |  |  |  |  | -6.92 | -13.92 | 0.07 | 0.052 | -6.89 | -14.77 | 0.98 | 0.086 |
| Maternal age (y) |  |  |  |  |  |  |  |  | 0.38 | 0.10 | 0.70 | 0.009 | 0.15 | -0.14 | 0.45 | 0.305 |
| Parity |  |  |  |  |  |  |  |  | -2.10 | -3.56 | -0.63 | 0.005 | -1.05 | -4.67 | 2.57 | 0.569 |
| Breastfed (never) |  |  |  |  |  |  |  |  | Referent | | | | Referent | | | |
| <1mo |  |  |  |  |  |  |  |  | 0.35 | -3.64 | 4.34 | 0.863 | -0.97 | -4.93 | 2.98 | 0.630 |
| 1 to <3mo |  |  |  |  |  |  |  |  | 1.79 | -2.23 | 5.81 | 0.382 | 0.70 | -3.27 | 4.67 | 0.729 |
| 3 to <6mo |  |  |  |  |  |  |  |  | 3.69 | -0.31 | 7.68 | 0.071 | 1.42 | -2.57 | 5.42 | 0.485 |
| ≥6 |  |  |  |  |  |  |  |  | 1.29 | -2.69 | 5.28 | 0.524 | -1.28 | -5.28 | 2.72 | 0.530 |
| No alcohol in pregnancy |  |  |  |  |  |  |  |  | Referent | | | | Referent | | | |
| 1-7 drinks/wk |  |  |  |  |  |  |  |  | -0.32 | -2.61 | 1.97 | 0.784 | -1.28 | -3.56 | 1.01 | 0.274 |
| >7 drinks/wk |  |  |  |  |  |  |  |  | 4.46 | -0.33 | 9.24 | 0.068 | 3.81 | -0.95 | 8.57 | 0.116 |
| Maternal pre-preg BMI |  |  |  |  |  |  |  |  | -0.38 | -0.68 | -0.08 | 0.012 | -0.38 | -0.67 | -0.08 | 0.012 |
| Partnered |  |  |  |  |  |  |  |  |  |  |  |  | 0.58 | -2.54 | 3.70 | 0.716 |
| Social Class (I & II) |  |  |  |  |  |  |  |  |  |  |  |  | Referent | | | |
| III |  |  |  |  |  |  |  |  |  |  |  |  | 0.22 | -2.30 | 2.73 | 0.866 |
| IV & V |  |  |  |  |  |  |  |  |  |  |  |  | 1.22 | -3.19 | 5.64 | 0.586 |
| Maternal Education (CSE + vocational) |  |  |  |  |  |  |  |  |  |  |  |  | Referent | | | |
| O level |  |  |  |  |  |  |  |  |  |  |  |  | 2.55 | -1.01 | 6.11 | 0.160 |
| A level |  |  |  |  |  |  |  |  |  |  |  |  | 1.38 | -2.58 | 5.34 | 0.494 |
| ≥Degree |  |  |  |  |  |  |  |  |  |  |  |  | 7.07 | 2.48 | 11.66 | 0.003 |
| Other children (none) |  |  |  |  |  |  |  |  |  |  |  |  | Referent | | | |
| 1 |  |  |  |  |  |  |  |  |  |  |  |  | -1.15 | -5.51 | 3.20 | 0.603 |
| ≥2 |  |  |  |  |  |  |  |  |  |  |  |  | -1.37 | -9.47 | 6.73 | 0.740 |
| Family Income (<£200) |  |  |  |  |  |  |  |  |  |  |  |  | Referent | | | |
| £ 200-399 |  |  |  |  |  |  |  |  |  |  |  |  | -0.96 | -4.40 | 2.48 | 0.583 |
| £ >400 |  |  |  |  |  |  |  |  |  |  |  |  | 2.39 | -1.00 | 5.77 | 0.166 |
| Maternal smoking (never) |  |  |  |  |  |  |  |  |  |  |  |  | Referent | | | |
| Quit |  |  |  |  |  |  |  |  |  |  |  |  | -1.13 | -3.58 | 1.33 | 0.367 |
| In pregnancy |  |  |  |  |  |  |  |  |  |  |  |  | -2.11 | -6.07 | 1.85 | 0.295 |
| HOME |  |  |  |  |  |  |  |  |  |  |  |  | 0.32 | -0.19 | 0.82 | 0.218 |
| Maternal IQ |  |  |  |  |  |  |  |  |  |  |  |  | 0.18 | 0.08 | 0.28 | <0.001 |

Abbreviations: CI, confidence interval; CSE Certificate of Secondary Education; IQ intelligence quotient

^1^ Eligible participants were children who had the Performance subscale of the Wechsler Intelligence Scale for Children measured at 8 years of age and had all dietary and covariable data.

^2^ Analyses show the beta-coefficient from multivariable linear regression analysis using generalized linear models. Model 1 is the association between full scale IQ at 8 years of age (outcome) and dietary pattern trajectory intercept and slope (predictors). Model 2: model 1 plus adjustment for all other dietary pattern trajectories and intercepts. Model 3: model 2 plus adjustment for the following perinatal variables; sex, gestational age at birth, birth weight, ethnicity, singleton/twin, maternal age and parity. Model 4: model 3 plus adjustment for the following sociodemographic variables; social class (according to standard UK classifications of occupation at the time of birth[3]), maternal education, other children, family income, maternal smoking, stimulation in the home environment (using an adaptation of the HOME questionnaire[4]) and maternal IQ (measured by WASI when the study child was 15 years of age).

^3^ Beta coefficients are scaled to reflect the change in dietary patterns over 6 to 24 months of age.

**Table S9:** Complete case analysis of the associations between dietary pattern trajectories measured from 6 to 24 months and full scale IQ^1^ at 15 years of age (n=956)^1^

|  | Model 1^2^ | | | | Model 2 | | | | Model 3 | | | | Model 4 | | | |
| --- | --- | --- | --- | --- | --- | --- | --- | --- | --- | --- | --- | --- | --- | --- | --- | --- |
|  | β^3^ | 95% CI | | p | β^3^ | 95% CI | | p | β^3^ | 95% CI | | p | β^3^ | 95% CI | | p |
|  |  | Lower | Upper |  |  | Lower | Upper |  |  | Lower | Upper |  |  | Lower | Upper |  |
| Healthy Intercept | 5.72 | 4.41 | 7.02 | <0.001 | 4.76 | 3.43 | 6.09 | <0.001 | 3.01 | 1.41 | 4.60 | <0.001 | 1.60 | 0.04 | 3.17 | 0.045 |
| Healthy slope | 1.72 | 0.61 | 2.82 | 0.002 | 1.03 | -0.09 | 2.14 | 0.071 | 0.75 | -0.37 | 1.87 | 0.187 | 0.16 | -0.92 | 1.24 | 0.771 |
| Discretionary Intercept | -6.14 | -9.18 | -3.11 | <0.001 | -5.65 | -8.69 | -2.60 | <0.001 | -3.29 | -6.44 | -0.15 | 0.040 | -1.42 | -4.46 | 1.61 | 0.357 |
| Discretionary slope | -2.49 | -3.53 | -1.46 | <0.001 | -1.85 | -2.90 | -0.80 | 0.001 | -1.36 | -2.42 | -0.31 | 0.011 | -0.73 | -1.76 | 0.29 | 0.159 |
| Traditional Intercept | 1.33 | -1.08 | 3.76 | 0.278 | 1.22 | -1.14 | 3.58 | 0.310 | 1.01 | -1.33 | 3.34 | 0.398 | 0.12 | -2.14 | 2.39 | 0.915 |
| Traditional slope | -14.10 | -28.31 | 0.11 | 0.052 | -0.58 | -14.65 | 13.49 | 0.935 | 0.49 | -13.43 | 14.42 | 0.945 | -1.26 | -14.60 | 12.07 | 0.853 |
| Ready-to-eat Intercept | 3.87 | -4.35 | 12.08 | 0.356 | 5.92 | -2.04 | 13.87 | 0.145 | 3.87 | -4.13 | 11.87 | 0.343 | -0.69 | -8.39 | 7.00 | 0.860 |
| Ready-to-eat slope | 26.72 | 6.13 | 47.31 | 0.011 | 17.31 | -2.73 | 37.34 | 0.090 | 11.24 | -9.10 | 31.58 | 0.279 | -2.27 | -21.90 | 17.36 | 0.821 |
| Female |  |  |  |  |  |  |  |  | -0.96 | -2.50 | 0.58 | 0.223 | -1.13 | -2.61 | 0.34 | 0.132 |
| Gestational age at birth (wk); 37-42 |  |  |  |  |  |  |  |  | Referent | | | | Referent | | | |
| <33 |  |  |  |  |  |  |  |  | -5.18 | -16.10 | 5.74 | 0.352 | -6.89 | -17.36 | 3.58 | 0.197 |
| 33-36 |  |  |  |  |  |  |  |  | 0.19 | -3.80 | 4.19 | 0.924 | -0.19 | -4.03 | 3.64 | 0.921 |
| >42 |  |  |  |  |  |  |  |  | 6.51 | -10.26 | 23.29 | 0.446 | 7.73 | -8.31 | 23.77 | 0.345 |
| Birth weight (kg) |  |  |  |  |  |  |  |  | 1.53 | -0.21 | 3.27 | 0.086 | 1.03 | -0.66 | 2.72 | 0.234 |
| White Ethnicity |  |  |  |  |  |  |  |  | 1.55 | -3.28 | 6.37 | 0.529 | 0.93 | -3.72 | 5.58 | 0.694 |
| Twin |  |  |  |  |  |  |  |  | -0.17 | -5.53 | 5.20 | 0.952 | 0.70 | -5.05 | 6.46 | 0.811 |
| Maternal age (y) |  |  |  |  |  |  |  |  | 0.42 | 0.21 | 0.63 | <0.001 | 0.17 | -0.04 | 0.38 | 0.114 |
| Parity |  |  |  |  |  |  |  |  | -2.06 | -3.11 | -1.01 | <0.001 | -0.11 | -2.59 | 2.38 | 0.934 |
| Breastfed (never) |  |  |  |  |  |  |  |  | Referent | | | | Referent | | | |
| <1mo |  |  |  |  |  |  |  |  | 1.30 | -1.53 | 4.12 | 0.368 | 0.07 | -2.66 | 2.79 | 0.962 |
| 1 to <3mo |  |  |  |  |  |  |  |  | 1.49 | -1.37 | 4.35 | 0.307 | 0.41 | -2.35 | 3.16 | 0.773 |
| 3 to <6mo |  |  |  |  |  |  |  |  | 4.73 | 1.87 | 7.59 | 0.001 | 2.24 | -0.54 | 5.01 | 0.114 |
| ≥6 |  |  |  |  |  |  |  |  | 4.39 | 1.51 | 7.27 | 0.003 | 1.74 | -1.07 | 4.54 | 0.225 |
| No alcohol in pregnancy |  |  |  |  |  |  |  |  | Referent | | | | Referent | | | |
| 1-7 drinks/wk |  |  |  |  |  |  |  |  | 1.32 | -0.35 | 2.99 | 0.121 | 0.21 | -1.41 | 1.84 | 0.799 |
| >7 drinks/wk |  |  |  |  |  |  |  |  | 2.50 | -0.94 | 5.93 | 0.154 | 1.64 | -1.67 | 4.95 | 0.332 |
| Maternal pre-preg BMI |  |  |  |  |  |  |  |  | -0.13 | -0.34 | 0.08 | 0.235 | -0.15 | -0.35 | 0.06 | 0.160 |
| Partnered |  |  |  |  |  |  |  |  |  |  |  |  | 0.07 | -2.07 | 2.21 | 0.949 |
| Social Class (I & II) |  |  |  |  |  |  |  |  |  |  |  |  | Referent | | | |
| III |  |  |  |  |  |  |  |  |  |  |  |  | -0.40 | -2.18 | 1.39 | 0.663 |
| IV & V |  |  |  |  |  |  |  |  |  |  |  |  | 1.53 | -1.57 | 4.62 | 0.333 |
| Maternal Education (CSE + vocational) |  |  |  |  |  |  |  |  |  |  |  |  | Referent | | | |
| O level |  |  |  |  |  |  |  |  |  |  |  |  | 2.71 | 0.20 | 5.21 | 0.034 |
| A level |  |  |  |  |  |  |  |  |  |  |  |  | 3.89 | 1.09 | 6.68 | 0.007 |
| ≥Degree |  |  |  |  |  |  |  |  |  |  |  |  | 7.01 | 3.74 | 10.29 | <0.001 |
| Other children (none) |  |  |  |  |  |  |  |  |  |  |  |  | Referent | | | |
| 1 |  |  |  |  |  |  |  |  |  |  |  |  | -1.27 | -4.28 | 1.75 | 0.410 |
| ≥2 |  |  |  |  |  |  |  |  |  |  |  |  | -4.11 | -9.69 | 1.46 | 0.148 |
| Family Income (<£200) |  |  |  |  |  |  |  |  |  |  |  |  | Referent | | | |
| £ 200-399 |  |  |  |  |  |  |  |  |  |  |  |  | 0.45 | -1.90 | 2.81 | 0.706 |
| £ >400 |  |  |  |  |  |  |  |  |  |  |  |  | 3.07 | 0.73 | 5.41 | 0.010 |
| Maternal smoking (never) |  |  |  |  |  |  |  |  |  |  |  |  | Referent | | | |
| Quit |  |  |  |  |  |  |  |  |  |  |  |  | -0.38 | -2.10 | 1.34 | 0.665 |
| In pregnancy |  |  |  |  |  |  |  |  |  |  |  |  | -0.08 | -2.88 | 2.72 | 0.953 |
| HOME |  |  |  |  |  |  |  |  |  |  |  |  | 0.23 | -0.12 | 0.58 | 0.197 |
| Maternal IQ |  |  |  |  |  |  |  |  |  |  |  |  | 0.20 | 0.13 | 0.26 | <0.001 |

Abbreviations: CI, confidence interval; CSE Certificate of Secondary Education; IQ intelligence quotient

^1^ Eligible participants were children who had IQ measured at 15 years of age using the Wechsler Abbreviated Scale of Intelligence and had all dietary and covariable data.

^2^ Analyses show the beta-coefficient from multivariable linear regression analysis using generalized linear models. Model 1 is the association between full scale IQ at 15 years of age (outcome) and dietary pattern trajectory intercept and slope (predictors). Model 2: model 1 plus adjustment for all other dietary pattern trajectories and intercepts. Model 3: model 2 plus adjustment for the following perinatal variables; sex, gestational age at birth, birth weight, ethnicity, singleton/twin, maternal age and parity. Model 4: model 3 plus adjustment for the following sociodemographic variables; social class (according to standard UK classifications of occupation at the time of birth[3]), maternal education, other children, family income, maternal smoking, stimulation in the home environment (using an adaptation of the HOME questionnaire[4]) and maternal IQ (measured by WASI when the study child was 15 years of age).

^3^ Beta coefficients are scaled to reflect the change in dietary patterns over 6 to 24 months of age.

**References**

1. Royston P (2005) Multiple imputation of missing values: update of ice. STATA J 5: 527-536.

2. North K, Emmet P, Avon Longitudinal Study of Pregnancy and Childhood (ALSPAC) Study Team (2000) Multivariate analysis fo diet amonth three-year-old children and associations with socio-demographic characteristics. Eur J Clin Nutr 54: 73-80.

3. Office of Population Censuses & Surveys (1991) Standard occupational classification. London: Her Majesty's Stationery Office.

4. Caldwell BM, Bradley RH (1979) Home observation for measurement of the environment. Little Rock: University of Arkansas.
